# Presentation, management, and clinical outcomes of acute type A dissection: Does sex matter?

**DOI:** 10.1016/j.xjon.2024.12.006

**Published:** 2024-12-16

**Authors:** Frederike Meccanici, Carlijn G.E. Thijssen, Arjen L. Gökalp, Marie H.E.J. van Wijngaarden, Mark F.A. Bierhuizen, Guy F. Custers, Jort Evers, Jolien A. de Veld, Maximiliaan L. Notenboom, Guillaume S.C. Geuzebroek, Joost F.J. ter Woorst, Jelena Sjatskig, Robin H. Heijmen, Mostafa M. Mokhles, Roland R.J. van Kimmenade, Jos A. Bekkers, Johanna J.M. Takkenberg, Jolien W. Roos-Hesselink

**Affiliations:** a Department of Congenital Cardiology, Erasmus MC, Rotterdam, The Netherlands; b Department of Cardiology, Radboud University Medical Center, Nijmegen, The Netherlands; c Department of Cardiothoracic Surgery, Erasmus MC, Rotterdam, The Netherlands; d Department of Cardiothoracic Surgery, Radboud University Medical Center, Nijmegen, The Netherlands; e Department of Cardiothoracic Surgery, Catharina Ziekenhuis, Eindhoven, The Netherlands; f Department of Cardiothoracic Surgery, St Antonius Ziekenhuis, Nieuwegein, The Netherlands; g Department of Cardiothoracic Surgery, Utrecht University Medical Center, Utrecht, The Netherlands

**Keywords:** Stanford type A aortic dissection, clinical presentation, outcomes, survival, sex, gender

## Abstract

**Background:**

Male–female differences in clinical presentation, management, and outcomes of acute type A aortic dissection (AD-A) have been reported; however, robust data are scarce. This study examined those differences.

**Methods:**

Consecutive adults diagnosed with AD-A between 2007 and 2017 in 4 referral centers were included retrospectively. Baseline data, operative characteristics, and mortality and morbidity during follow-up were collected using patient files, questionnaires, and referral information.

**Results:**

The study included 889 patients (37.5% female). Females were significantly older at presentation (median, 67.0 [interquartile range [IQR], 59.0-75.0] years vs 61.0 [IQR, 53.0-69.0] years; *P* < .001) and more often had cardiovascular comorbidities. Severe hypotension, tamponade, and nausea were more frequently observed in females. Short-term mortality was 18.5% in females and 21.2% in males (*P* = .362). No significant differences in treatment between males and females were observed. After surgery, the median follow-up was 6.2 years (IQR, 3.5-9.2 years). Overall 10-year survival was 50.1% (95% confidence interval [CI], 43.6%-57.6%) in females and 62.8% (95% CI, 58.1%-67.9%) in males (*P* = .009), although this difference was not significant after multivariable correction. Compared to the matched general population, survival was lower than expected in females and comparable to expected in males. The long-term reintervention rate in surgically treated survivors was comparable between males and females (2.1%/patient-year). Male- and female-specific risk factors for long term mortality were identified.

**Conclusions:**

These findings highlight a distinct clinical profile at presentation with AD-A between males and females, while treatment approach and short-term mortality were comparable. The relatively poor long-term survival in females and male-/female-specific risk stratification warrant further investigation.

**Figure:**
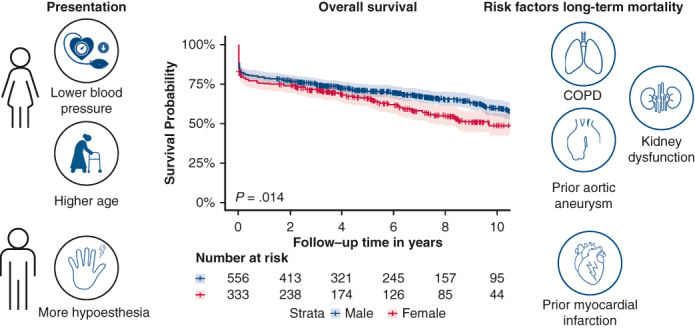
Observed male–female differences in AD-A patients.

**Central Message**

Despite distinct clinical profiles, no male–female differences in short-term mortality for AD-A patients were observed. After surgery, unadjusted long-term survival was worse for females.

**Perspective**

Sex-specific differences in clinical presentation of acute type A aortic dissection (AD-A) are evident, though treatment strategies and short-term mortality are comparable. The relatively poor long-term survival in females underscores the need for further research into sex-based risk stratification and management.

Acute Stanford type A aortic dissection (AD-A) is a cardiovascular emergency with an estimated incidence of 12 cases per 100,000 persons per year and a male:female ratio of 2:1.^1^^,^^2^ The incidence has increased over the past several decades and often is underestimated due to high prehospital mortality.^1^ After diagnosis, AD-A carries a high 30-day mortality of 20% to 40%.^3^, ^4^, ^5^ However, this might be an underestimation of the total short-term mortality, because AD-A has considerable prehospital mortality.

Emergency surgery is the cornerstone of AD-A treatment, and thus rapid diagnosis of AD-A is essential.^6^ In recent decades, surgical intervention has improved the short-term prognosis of AD-A considerably^3^^,^^7^; however, AD-A is a complex disease, and even with successful repair, the risk of postoperative morbidity and recurrent aortic events remains high.^8^^,^^9^ Therefore, exploring factors associated with long-term outcomes and quality of life in these patients is crucial.

Insights into patient-specific factors associated with outcomes after AD-A can improve risk stratification and help optimize tailored treatment strategies. Attention has been drawn to male–female differences in presentation, treatment, outcomes, and mortality that have been reported. The findings have been contradictory; although some studies have reported higher mortality in females,^7^^,^^10^^,^^11^ more recent studies, including a meta-analysis, have indicated comparable short-term mortality in males and females.^12^^,^^13^ Most of these studies have involved small, single-centre populations with limited follow-up, however. Large registries focus primarily on mortality and often lack detailed clinical outcomes and questionnaires.^14^ Consequently, knowledge about male–female differences in clinical outcomes of AD-A is limited, especially regarding long-term follow-up. This knowledge is essential to enable male- and female-specific recommendations in guidelines for thoracic aortic aneurysms and dissection.^6^^,^^15^^,^^16^

We performed this multicenter retrospective cohort study of patients diagnosed with AD-A in 4 large aortic surgery centers in the Netherlands to explore male–female differences in clinical presentation, disease management, and clinical outcomes.

## Methods

### Study Population

Data were collected on all adult (age ≥18 years at diagnosis) patients who presented with AD-A at 4 large aortic surgery centers in The Netherlands between January 1, 2007, and December 31, 2017. Exclusion criteria were non–AD-A, asymptomatic patients in whom the time of symptom onset could not be determined, and iatrogenic dissection or aortic dissection secondary to trauma. Participating centers included Erasmus Medical Centre (Rotterdam, The Netherlands), Radboud University Medical Centre (Nijmegen, The Netherlands), Catharina Ziekenhuis (Eindhoven, The Netherlands), and St Antonius Ziekenhuis (Nieuwegein, The Netherlands).

This study was approved by the local Ethics Committee (MEC-2018-1535; approved November 12, 2018) of each participating center with a waiver of individual consent and was designed, performed, and controlled in accordance with current local and international good clinical practice guidelines.

### Data Collection

Patients were identified using the institutional aortic surgery databases. Additionally, an extensive search was performed using the hospitals' diagnosis registration systems. Digital files of all patients with diagnosis treatment codes related to any thoracic aortic disease were screened manually to identify patients eligible for inclusion. Data were collected using a standardized case report form with an electronic Clinical Research Form application (OpenClinica, version 3.6). The case report form included information on patients' demographics, medical history, clinical presentation, imaging findings, surgical procedures, postsurgical complications, and mortality. Variables and their definitions are provided in Online Data Supplement. Information on survival was obtained from Dutch municipal registries. Data on long-term morbidity were obtained from the hospitals’ digital patient files. Additionally, questionnaires were sent to all survivors that contained questions on long-term morbidity based on the Akins guidelines.^17^ In advance of sending out the questionnaire, a mortality check was performed using the Dutch municipal registries. Between July 2019 and February 2021, the questionnaire, along with an informed consent form, was sent to all AD-A survivors. If a (potential) event was reported in the questionnaire, additional data were requested from the treating physician to verify the event.

### Definitions

Sex was defined as biological, meaning the sex at birth. “Acute” was defined as presentation within 14 days of symptom onset according to current guidelines.^6^ Short-term mortality and morbidity events were defined as events occurring within 30 days from the operation or diagnosis date or occurring before hospital discharge. Severity of valve regurgitation or valve stenosis was determined by echocardiography and categorized as none, grade 1 (minimal), grade 2 (mild), grade 3 (moderate) and grade 4 (severe). Body surface area (BSA) was calculated using the DuBois and DuBois formula.^18^ Severe hypotension was defined as systolic blood pressure <90 mm Hg or medical treatment for severe hypotension at presentation or before surgery. Normal values for laboratory results were calculated using the Dutch Association for Laboratory Medicine's reference values.^19^ Mortality was described for the total cohort, including short-term mortality (overall mortality), the total cohort excluding short-term mortality (long-term mortality), and the total cohort excluding preoperative deaths only (surgical mortality). The time to an event was calculated from the date of operation or diagnosis to the time of the event, time of the mortality check, or censoring.

### Statistical Analyses

Data analyses were performed with statistical and computing program R version 3.6.1 (R Foundation for Statistical Computing). Normality was checked visually with histograms and tested using the Shapiro-Wilk test. Descriptive analyses were used for the patient and procedural characteristics. The Student *t* test was used to compare normally distributed continuous data, and data were presented as mean and standard deviation (SD). The Mann-Whitney *U* test was used to compared skewed continuous data, and the data are presented as median and interquartile range (IQR). For categorical data, the χ^2^ test or Fisher exact test was used, and the data are presented as percentages or frequencies. For the postoperative 30-day outcomes, the events are presented as percentage of the total patient population and compared using the χ^2^ test or Fisher exact test.

For short-term mortality, univariable and multivariable logistic regression analysis was performed including only patients who were surgically treated for AD-A. Preoperative and perioperative variables were tested first in a univariable model for the total cohort and for males and females separately. The variables with a *P* value < .10 in univariable analysis without significant collinearity (Pearson correlation coefficient <0.7) were entered in a backward selection process, applying a threshold *P* value < .10. Multivariable logistic regression analysis was adjusted considering the 4 different study centers. Odds ratios (ORs) with their corresponding 95% confidence intervals (CIs) are presented. In addition, the expected mortality as calculated by the Logistic EuroSCORE was compared to the observed short-term mortality in our cohort.

Completeness of follow-up was calculated using the modified Clark method.^20^ For the description of long-term outcomes, both time-to-event and linearized occurrence rates were computed. For mortality and long-term reintervention, Kaplan-Meier estimates were constructed and compared between males and females with the log-rank test or the Peto & Peto modification of the Gehan-Wilcoxon test, as appropriate. In addition, the cumulative incidence of any thoracic aortic reintervention was assessed in the presence of death as a competing risk through the Aalen-Johansen estimator to generate sex-specific multistate curves, using the R packages *tidycompr* and *cuminc*. Gray's test was used to evaluate hypotheses of equality of cause-specific cumulative incidence functions between males and females.

All long-term events are shown as linearized occurrence rates in percentage per patient-year (95% CI): the total number of events divided by the total follow-up in patient-years. Rate ratios were calculated to compare occurrence rates between females and to males. Additionally, long-term events were depicted as the 10-year event-free probability and corresponding 95% CI, and hazard ratios (HRs) with 95% CIs comparing females to males were calculated.

The long-term survival of AD-A patients excluding short-term mortality was compared with that of an estimated matched general population survival using individual-level parameters (matched by sex, exact age and calendar year of each individual, and censoring time) using an application developed by Wang and colleagues.^21^ Kaplan-Meier estimates of the matched general population survival were constructed for the total study population and for females and males separately.

For long-term mortality and reintervention, separate risk factor analyses were performed using Cox proportional hazards models, with stratification of the baseline hazard by study center. Only patients who survived to hospital discharge and >30 days were included in these analyses. Patient characteristics and the operative procedure were first tested in a univariable model for the total cohort and males and females separately. For the multivariable Cox regression analysis, the same methods for backward selection were used as reported for short-term mortality. The results of the Cox regression analyses are presented as HR and 95% CI.

The missing data pattern of patient characteristics and operative procedure was assumed to be missing (completely) at random. Imputation of variables used in both the logistic regression analyses and the Cox regression analyses was performed up to a 15% rate of missing values. The multiple imputation was performed using the *mice* package with 5 imputed datasets and 10 iterations, and the 5 imputed datasets were pooled using Rubin's rules. A 2-sided *P* value < .05 was considered statistically significant.

## Results

### Clinical Presentation and Surgical Characteristics

A total of 889 patients were included (331 females; 37.2%). Male–female differences in baseline characteristics are shown in Table 1. The median patient age was 63.0 years [IQR, 55.0-71.0 years], and female patients were significantly older than male patients (median, 67.0 [IQR, 59.0-75.0] years vs 61.0 [IQR, 53.0-69.0] years; *P* < .001). Online Data Supplement summarizes male–female differences in clinical presentation, including Online Data Supplement which shows symptoms reported by male and female patients at AD-A presentation. Females reported nausea significantly more often than males (71.2% vs 57.2%; *P* = .024), whereas males reported hypoesthesia more often (44.1% vs 29.3%; *P* = .008). Time from symptom onset to diagnosis was not significantly different between males and females (*P* = .208). Online Data Supplement compares diagnostic imaging and laboratory results in males and females.

**Table 1:** Patient baseline characteristics

Characteristic	Total (N = 889)	Males (N = 558)	Females (N = 331)	*P* value	Missing, %
Age, y, median [IQR]	63.0 [55.0-71.0]	61.0 [53.0-69.0]	67.0 [59.0-75.0]	<.001∗	0.2
Body surface area, m^2^, mean ± SD	2.0 ± 0.2	2.1 ± 0.2	1.8 ± 0.2	<.001∗	30.5
History of hypertension, n (%)	425 (50.8)	241 (46.0)	184 (59.0)	<.001∗	6.0
History of diabetes mellitus, n (%)	19 (2.2)	7 (1.3)	12 (3.8)	.034†	3.3
History of COPD, n (%)	64 (7.4)	34 (6.3)	30 (9.3)	.133	3.0
History of CVA, n (%)	41 (4.8)	23 (4.3)	18 (5.6)	.461	3.4
History of chronic kidney disease, n (%)	25 (2.9)	10 (1.8)	15 (4.7)	.028†	3.3
Smoking ≥1 pack-y, n (%)				.116	58.3
Currently	190 (51.2)	124 (51.5)	66 (50.8)		
In past	78 (21.0)	57 (23.7)	21 (16.2)		
History of dyslipidemia, n (%)	93 (10.8)	51 (9.5)	41 (13.0)	.148	4.3
History of myocardial infarction, n (%)	41 (4.8)	25 (4.6)	16 (5.0)	.961	3.3
Prior aortic surgery, n (%)	27 (3.1)	20 (3.7)	7 (2.2)	.294	3.1
Prior cardiac surgery, n (%)				.292‡	2.7
CABG	14 (1.6)	11 (2.0)	3 (0.9)		
MVR	6 (0.7)	6 (1.1)	0 (0.0)		
CABG + MVR	3 (0.3)	3 (0.6)	0 (0.0)		
PCI	14 (1.6)	10 (1.8)	4 (1.2)		
Other	7 (0.8)	4 (0.7)	3 (0.9)		
Aortic valve stenosis, n (%)§	7 (2.5)	4 (2.5)	3 (2.6)	1.000	68.5
Aortic valve regurgitation, n (%)§	18 (6.4)	12 (7.4)	6 (5.0)	.589	68.3
Mitral valve regurgitation, n (%)	10 (3.6)	8 (4.9)	2 (1.7)	.268	68.4
Thoracic aortic aneurysm, n (%)	70 (8.2)	37 (6.9)	33 (10.3)	.103	4.0
Last measured aortic diameter, mm, median [IQR]	47.4 [44.3-50.0]	47.9 [45.3-50.0]	47.0 [43.0-50.0]	.387	93.0
Last measured diameter indexed for BSA, mm/m^2^, median [IQR]	23.7 [22.6-26.9]	22.8 [21.9-24.2]	26.8 [23.7-28.9]	<.001∗	94.4
Prior dissection or aneurysm in other arteries, n (%)	35 (4.1)	26 (4.9)	9 (2.8)	.198	3.6
Bicuspid aortic valve, n (%)	21 (2.7)	15 (3.1)	6 (2.0)	.483	13.0
Presence of connective tissue disease, n (%)‖	31 (10.2)	19 (9.6)	12 (11.3)	.881	65.9

*IQR*, Interquartile range; *COPD*, chronic obstructive pulmonary disease; *CVA*, cerebrovascular accident (including transient ichemic attack); *CABG*, coronary artery bypass grafting; *MVR*, mitral valve replacement; *PCI*, percutaneous coronary intervention; *BSA*, body surface area.

∗ Significant at the .01 level.

† Significant at the .05 level.

‡ Fisher exact test.

§ Defined as at least moderate stenosis/regurgitation.

‖ Confirmed connective tissue diseases: Marfan syndrome (7 patients), *ACTA2* mutation (6 patients), Loeys-Dietz syndrome (3 patients), Turner syndrome (3 patients), Ehlers-Danlos syndrome (1 patient), and other mutations associated with connective tissue disorders (8) patients.

Surgery was performed in 878 patients (98.8%), including 551 males (98.7%) and 327 females (98.8%; *P* = 1.00), as shown in Table 2. Eleven patients were managed nonsurgically; 8 patients died before surgery or were declined because of severe hemodynamic instability or cardiac arrest, 1 patient was declined because of refusal of blood transfusion (Jehovah's Witness), 1 patient was declined due to significant comorbidities (and life expectancy <1 year), in 1 patient the reason for not performing surgery was unknown. No significant male–female differences in medical and surgical treatment characteristics were observed. Ascending aortic surgery was performed in 850 patients (97.7%); 20 patients did not receive ascending aortic surgery, and in 8 patients data were missing. Of the 20 patients who did not undergo ascending aortic surgery, 8 underwent only aortic valve and root surgery, 2 underwent only aortic arch replacement, and in 10 patients surgery was initiated but discontinued because of hemodynamic instability/resuscitation setting.

**Table 2:** Treatment of males and females with AD-A

Treatment	All (N = 889), n (%)	Males (N = 558), n (%)	Females (N = 331), n (%)	*P* value	Missing, %
Antihypertensive medication	124 (26.3)	80 (27.8)	44 (23.9)	.410	46.9
Diuretics	5 (1.2)	2 (0.8)	3 (1.8)	.622	51.6
Vasodilators	146 (31.9)	94 (33.9)	52 (28.6)	.304	48.6
Surgery performed	878 (98.8)	551 (98.7)	327 (98.8)	1.000	0.0
Ascending aortic surgery	847 (97.7)	532 (98.2)	315 (96.9)	.692	2.6
Aortic arch surgery					2.4
Partial or total arch replacement	136 (15.9)	91 (16.9)	45 (14.1)	.264	
Hemi-arch	425 (49.6)	275 (51.2)	150 (46.9)	.220	
Repair	16 (1.9)	11 (2.0)	5 (1.6)	.611	
Elephant trunk	1 (0.1)	1 (0.2)	0 (0.0)	.440	
Descending aortic surgery	10 (1.1)	7 (1.3)	3 (0.9)	.879	2.5
Concomitant procedures	66 (7.4)	40 (7.3)	26 (7.9)	.643	0.0
CABG	55 (6.2)	31 (5.6)	24 (7.3)		
MV surgery	2 (0.2)	1 (0.2)	1 (0.3)		
Other	10 (1.1)	8 (1.4)	2 (0.6)		
Types of aortic valve surgery					
Bentall procedure				.229	3.1
Mechanical	105 (12.2)	72 (13.3)	33 (10.3)		
Biological	46 (5.3)	25 (4.6)	21 (6.5)		
VSARR procedure				.430	3.1
David	11 (1.3)	7 (1.3)	4 (1.2)		
(Partial) Yacoub	1 (0.1)	0 (0.0)	1 (0.3)		
Supracoronary replacement				.512	3.1
No AVR	328 (37.9)	201 (37.2)	127 (39.6)		
Mechanical AVR	25 (2.9)	18 (3.3)	7 (2.2)		
Biological AVR	26 (3.0)	19 (3.5)	7 (2.2)		
Valve repair	295 (34.3)	180 (33.3)	115 (35.8)		

*AD-A*, Acute thoracic aortic dissection Stanford type A; *CABG*, coronary artery bypass grafting; *MV*, mitral valve; *VSARR*, valve-sparing aortic root replacement; *AVR*, aortic valve replacement.

### Short-Term Mortality and Morbidity

Postoperative mortality and morbidity rates are shown in Table 3. Short-term mortality was 19.5% overall (n = 171/889) and was not significantly different between females (20.5%; n = 68/331) and males (18.5%; n = 103/558) (*P* = .50). In patients who underwent surgery for AD-A (n = 878; 98.8%) short-term mortality (surgical mortality) was 18.3% overall (17.4% in females vs 19.6% in males; *P* = .426). All nonoperated patients (n = 11; 1.2%) died in hospital within 1 to 3 days after diagnosis. Online Data Supplement provides the results of univariable and multivariable logistic regression analyses for short-term mortality, excluding nonsurgical patients, for the total cohort and stratified by sex. The final model in multivariable analysis included age (OR, 1.04; 95% CI, 1.02-1.06; *P* < .001), chronic obstructive pulmonary disease (COPD; OR, 2.89; 95% CI, 1.57-5.31; *P* < .001), descending aortic surgery (OR, 6.76; 95% CI, 1.69-27.1; *P* = .007) and concomitant procedures (OR, 4.25; 95% CI, 2.44-7.41; *P* < .001) (Online Data Supplement). Due to the limited number of events, no stratified multivariable analysis was performed for males and females separately. Online Data Supplement shows the observed versus expected mortality as calculated by the Logistic EuroSCORE in males and females.

**Table 3:** Short-term mortality and morbidity after diagnosis

Parameter	All (N = 889)	Male patients (N = 558)	Female patients (N = 331)	*P* value	Missing, %
Short-term mortality, n (%)	171 (19.5)	103 (18.5)	68 (20.5)	.500	0.0
Cause of death, n (%)				.272	1.2
Cardiac	46 (5.2)	31 (5.6)	15 (4.6)		
Neurologic	42 (4.8)	21 (3.8)	21 (6.4)		
Organ failure	22 (2.5)	14 (2.5)	8 (2.4)		
Aortic rupture	8 (0.9)	4 (0.7)	4 (1.2)		
Bleeding	16 (1.8)	12 (2.2)	4 (1.2)		
Sepsis	11 (1.2)	4 (0.7)	7 (2.1)		
Other	18 (2.0)	12 (2.2)	5 (1.5)		
Hospital stay, d, median [IQR]	13.0 [8.0-23.0]	14.0 [9.0-24.0]	13.0 [8.0-23.0]	.475	0.4
ICU stay, d, median [IQR]	5.0 [3.0-10.0]	5.0 [3.0-11.0]	5.0 [3.0-9.0]	.406	6.9
Ventilatory support, d, median [IQR]	2.0 [2.0-5.0]	2.0 [2.0-5.0]	2.0 [2.0-5.0]	.836	10.1
Early reoperation, n (%)	257 (29.8)	174 (32.2)	83 (25.8)	.054	3.0
Indication for reoperation, n (%)				.227	3.6
Bleeding event	170 (19.7)	116 (21.5)	54 (16.8)		
Tamponade	16 (1.9)	10 (1.9)	6 (1.9)		
Mediastinitis	8 (0.9)	6 (1.1)	2 (0.6)		
Valve dysfunction	2 (0.2)	0 (0.0)	2 (0.6)		
Other	37 (4.3)	25 (4.6)	12 (3.7)		
Gauze removal	18 (2.1)	14 (2.6)	4 (1.2)		
Infection, n (%)	275 (32.4)	167 (31.4)	108 (34.0)	.484	4.8
Bleeding, n (%)	208 (24.5)	142 (26.6)	66 (20.8)	.068	4.8
Sepsis, n (%)	61 (7.2)	43 (8.1)	18 (5.7)	.230	5.0
MI or ischemia, n (%)	15 (1.8)	8 (1.5)	7 (2.2)	.640	4.8
CVA after surgery, n (%)	94 (11.2)	55 (10.5)	39 (12.4)	.457	5.8
TIA after surgery, n (%)	8 (0.9)	3 (0.6)	5 (1.6)	.161∗	5.5
Device implantation, n (%)	11 (1.3)	6 (1.1)	5 (1.6)	.811	4.6
Spinal cord lesion, n (%)	15 (1.8)	12 (2.2)	3 (0.9)	.188∗	4.4
Lowest eGFR, median [IQR]	47.0 [28.0-60.0]	46.0 [25.0-60.0]	50.0 [32.0-60.0]	.042†	18.1

Short-term mortality defined as intraoperative or postoperative death <30 days or during the hospital stay. Early reoperation defined as reoperation <30 days or during the hospital stay. *IQR*, Interquartile range; *ICU*, intensive care unit; *MI*, myocardial infarction; *CVA*, cerebrovascular accident; *TIA*, transient ischemic attack; *eGFR*, estimated glomerular filtration rate.

∗ Fisher exact test.

† Significant at the .05 level.

### Follow-up Mortality and Morbidity

A flow chart of patient selection for long-term outcomes is depicted in Online Data Supplement. The median duration of follow-up was 6.17 years (IQR, 3.53-9.23 years; range, 0.17-14.0 years) for the total cohort, 5.74 years (IQR, 3.51-8.71 years; range, 0.19-13.7 years) for females and 6.34 years (IQR, 3.64-9.48 years; range, 0.17-14.0 years) for males (*P* = .161). The long-term mortality status could not be ascertained for 4 patients. Follow-up completeness for survival was 99.1% (4592 patient-years observed out of 4632 possible patient-years). This translates to 40 unobserved follow-up years due to dropout. The questionnaires on morbidity were sent to 555 patients who were alive, and 326 questionnaires (58.7%) were returned. Online Data Supplement presents the characteristics of the cohort of 711 AD-A survivors for the total cohort and also stratified for males and females.

**Figure 1:**
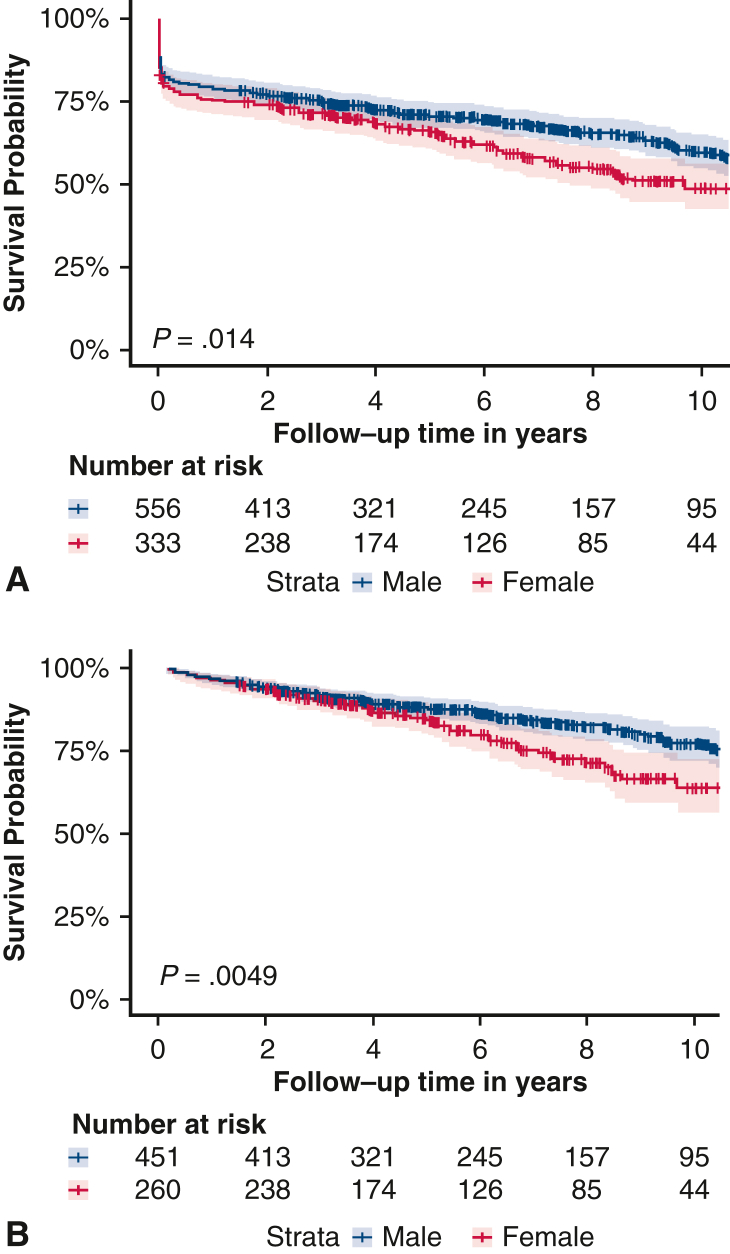
Kaplan-Meier estimates including 95% confidence intervals for overall survival including short-term mortality (A) and long-term survival excluding short-term mortality (B) stratified by sex.

Figure 1 presents the Kaplan-Meier estimates for overall mortality and long-term mortality excluding short term mortality stratified by sex. Kaplan-Meier estimates for surgical mortality are depicted in Online Data Supplement.

In Table 4, linearized occurrence rates for mortality and cardiovascular events during follow-up are reported for the total cohort and for males and females separately. Online Data Supplement presents the time-to-event outcomes are presented as 10-year event-free probability for all long-term cardiovascular events. Online Data Supplement shows the causes of death (if known), New York Heart Association class, and specifications of pacemaker or implantable cardioverter-defibrillator implantation during follow-up.

**Table 4:** Cardiovascular events during long-term follow-up in AD-A patients for the total cohort and stratified for males and females

Event	Total population		Females		Males		Female/male rate ratio (95% CI)	*P* value (Wald)
	Patients/events	% per PTY (95% CI)	Patients/events	% per PTY (95% CI)	Patients/events	% per PTY (95% CI)		
Overall mortality	317	6.87 (6.14-7.67)	137	8.46 (7.10-10.0)	180	6.01 (5.17-6.96)	1.41 (1.12-1.76)	.002
Long-term mortality	143	3.10 (2.62-3.66)	55	4.08 (3.15-5.19)	77	2.58 (2.03-3.22)	1.58 (1.14-2.20)	.006
Long-term reintervention∗	81/98	2.13 (1.73-2.59)	26/34	2.10 (1.46-2.94)	55/64	2.14 (1.64-2.73)	0.98 (0.64-1.48)	.929
Proximal	34/36	0.78 (0.55-1.08)	13/14	0.87 (0.47-1.45)	21/22	0.74 (0.46-1.11)	1.18 (0.59-2.29)	.636
Distal	62/73	1.58 (1.24-1.99)	21/25	1.55 (1.00-2.28)	41/48	1.61 (1.18-2.13)	0.97 (0.59-1.55)	.876
Thoracic aortic aneurysm	58/62	1.35 (1.03-1.73)	17/20	1.24 (0.76-1.91)	41/42	1.41 (1.01-1.90)	0.88 (0.51-1.49)	.637
False aneurysm	22/23	0.50 (0.32-0.75)	11/12	0.74 (0.38-1.30)	11/11	0.37 (0.18-0.66)	2.01 (0.87-4.68)	.087
Abdominal aortic aneurysm	13/14	0.30 (0.17-0.51)	1/1	0.06 (0.00-0.34)	12/13	0.44 (0.23-0.74)	0.16 (0.01-0.81)	**.028**
Thoracic aortic dissection	5/5	0.11 (0.04-0.25)	2/2	0.12 (0.02-0.45)	3/3	0.10 (0.02-0.29)	1.26 (0.15-8.28)	.819
Myocardial infarction	5/5	0.11 (0.04-0.25)	0/0	0.0	5/5	0.17 (0.05-0.39)	-	.100
CVA	29/35	0.76 (0.53-1.06)	6/6	0.37 (0.14-0.81)	23/29	0.97 (0.65-1.39)	0.39 (0.14-0.88)	**.026**
TIA	31/32	0.70 (0.48-0.98)	8/9	0.56 (0.25-1.06)	23/23	0.77 (0.49-1.15)	0.73 (0.32-1.53)	.407
Bleeding	5/5	0.11 (0.04-0.25)	0/0	0.0	5/5	0.17 (0.05-0.39)	-	.100
Endocarditis	15/15	0.33 (0.18-0.54)	4/4	0.25 (0.07-0.63)	11/11	0.37 (0.18-0.66)	0.69 (0.18-2.04)	.493
Operative valve dysfunction†	7/7	0.27 (0.11-0.56)	2/2	0.23 (0.03-0.83)	5/5	0.30 (0.10-0.69)	0.77 (0.10-3.75)	.717

The number of patients with an event and the total number of events are depicted for the total cohort and males and females separately. The linearized occurrence rates are presented as percentage per patient year with corresponding 95% confidence interval (CI), and females and males are compared using a rate ratio with 95% CI. *P* values < .05 are depicted in bold type. *AD-A*, Acute thoracic aortic dissection Stanford type A; *PTY*, patient-year; *CI*, confidence interval; *CVA*, cerebrovascular accident; *TIA*, transient ischemic attack.

∗ Considered proximal if the ascending aorta, aortic root or aortic valve were involved and distal if the aortic arch or descending aorta were involved in the procedure.

† Only for patients who underwent aortic valve replacement or repair at AD-A surgery.

**Figure 2:**
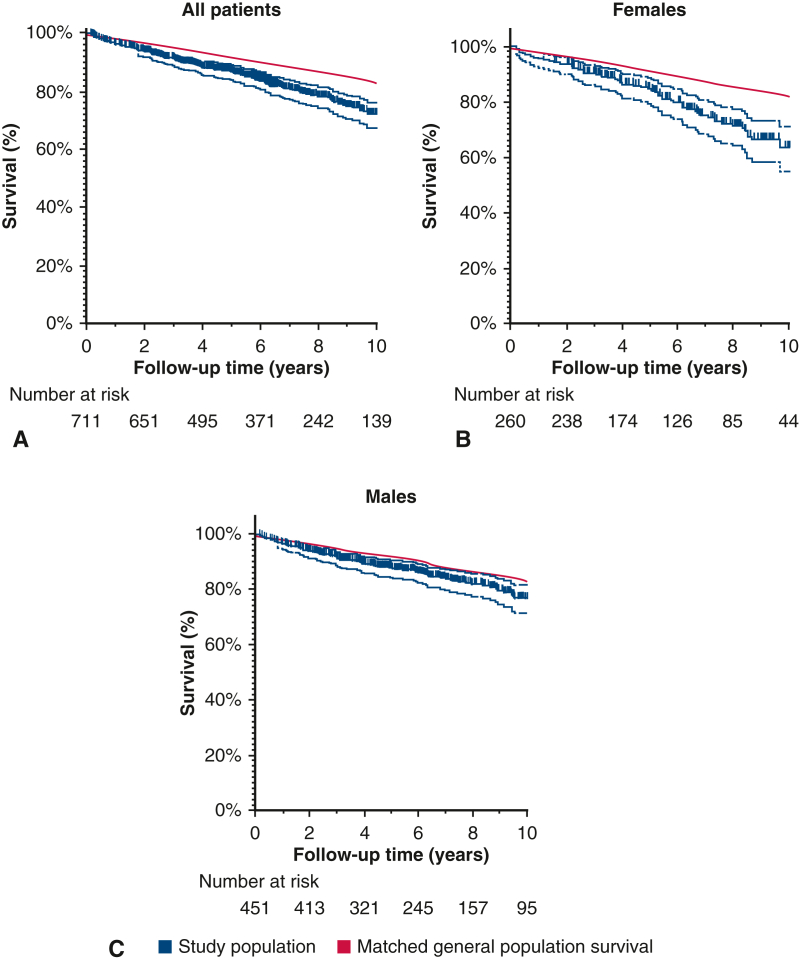
Kaplan-Meier survival estimates including 95% confidence intervals compared to the matched general population survival for the total cohort (A), females (B), and males (C). It was not possible to calculate the numbers at risk for the matched general population.

Figure 2 shows the Kaplan-Meier estimates of the AD-A study population for long-term mortality (excluding short-term mortality) together with the expected matched general population survival for the total cohort and for females and males separately.

In Online Data Supplement, the cumulative incidence function for reintervention with death as competing risk and the Kaplan-Meier estimates for freedom from reintervention are shown. The 10-year cumulative incidence for reintervention was 17.0% (95% CI, 10.9%-24.2%) for females and 17.2% (95% CI, 12.8%-22.0%) for males (*P* = .40). The 10-year freedom from reintervention was 80.5% (95% CI, 72.8%-89.0%) for females and 82.0% (95% CI, 77.2%-87.1%) for males (*P* = .51).

### Risk Factor Analyses for Late Mortality and Reintervention

Table 5 shows the results of univariable and multivariable Cox regression analysis for long-term mortality on the imputed dataset. Online Data Supplement presents all tested variables in univariable analysis for long-term mortality in the complete-case analysis.

**Table 5:** Cox regression analysis for long-term mortality after AD-A for all patients excluding short term mortality and stratified by sex

Variable	All patients (n = 711)				Females (n = 260)				Males (n = 451)			
	Univariable analysis		Multivariable analysis		Univariable analysis		Multivariable analysis		Univariable analysis		Multivariable analysis	
	HR (95% CI)	*P* value	HR (95%CI)	*P* value	HR (95%CI)	*P* value	HR (95%CI)	*P* value	HR (95%CI)	*P* value	HR (95% CI)	*P* value
Demographics												
Female sex (ref: male)	1.58 (1.13-2.21)	**.007**∗	-	-	-	-	-	-	-	-	-	-
Age (per 1 y increase)	1.07 (1.05-1.09)	**<.001**∗	1.07 (1.05-1.09)	**<.001**	1.07 (1.04-1.10)	**<.001**∗	1.07 (1.04-1.11)	**<.001**	1.06 (1.04-1.09)	**<.001**∗	1.06 (1.04-1.09)	**<.001**
History of hypertension	1.33 (0.94-1.88)	.105∗	-	-	1.21 (0.71-2.08)	.482∗	-	-	1.33 (0.84-2.12)	.222	-	-
History of hyperlipidemia	1.17 (0.69-1.99)	.562	-	-	1.21 (0.57-2.59)	.619	-	-	1.16 (0.55-2.44)	.702	-	-
Diabetes mellitus	1.65 (0.60-4.56)	.330	-	-	1.75 (0.52-5.85)	.358	-	-	0.80 (0.10-6.18)	.826	-	-
COPD	1.85 (1.04-3.30)	**.036**∗	-	-	2.58 (1.28-5.17)	**.009**∗	2.18 (1.07-4.45)	**.033**	0.91 (0-2.96)	.872	-	-
Prior CVA	1.86 (0.97-3.59)	.063∗	-	-	1.45 (0.56-3.76)	.443	-	-	2.39 (0.93-6.12)	.068∗	-	-
Prior MI	2.58 (1.49-4.47)	**<.001**∗	2.11 (1.20-3.72)	**.010**	1.79 (0.73-4.35)	.197	-	-	2.98 (1.40-6.32)	**.005**∗	2.40 (1.13-5.11)	**.023**
Chronic kidney disease	3.56 (1.79-7.10)	**<.001**∗	2.44 (1.18-5.04)	**.017**	3.21 (1.35-7.64)	**.009**∗	4.11 (1.68-10.1)	**.003**	3.62 (1.08-12.1)	**.037**∗	-	-
History of thoracic aortic aneurysm	2.22 (1.30-3.79)	**.004**∗	-	**-**	3.26 (1.53-6.95)	**.003**∗	2.89 (1.33-6.28)	**.008**	2.04 (0.91-4.55)	.081∗	-	-
Prior aortic surgery	2.99 (1.48-6.03)	**.002**∗	2.49 (1.22-5.08)	**.013**	6.74 (2.32-19.6)	**<.001**∗	-	-	2.45 (0.94-6.36)	.066∗	-	-
Prior cardiac surgery	1.78 (0.95-3.34)	.070	-	-	2.35 (0.82-6.72)	.110	-	-	1.61 (0.72-3.60)	.241	-	-
Bicuspid aortic valve	1.22 (0.49-3.04)	.662	-	-	2.04 (0.44-9.46)	.354	-	-	1.33 (0.39-4.51)	.640	-	-
Aortic valve surgery	1.11 (0.76-1.62)	.584	-	-	0.75 (0.44-1.30)	.299	-	-	1.80 (1.04-3.13)	**.040**∗	1.75 (1.00-3.04)	.050
Aortic arch surgery	0.98 (0.66-1.45)	.921	-	-	0.85 (0.49-1.47)	.557	-	-	1.27(0.69-2.31)	.437	-	-

Univariable and multivariable Cox regression analysis for all patients and females and males separately are shown. *P* values < .05 are depicted in bold type. “-“ denotes that the variable was not included in the multivariable model. *AD-A*, Acute thoracic aortic dissection Stanford type A; *HR*, hazard ratio; *CI*, confidence interval; *COPD*, chronic obstructive pulmonary disease; *CVA*, cerebrovascular accident; *MI*, myocardial infarction.

∗ Entered in full model for backward selection of the final multivariable model.

Online Data Supplement presents the results of univariable Cox regression analysis for late reintervention. For the total cohort, aortic arch surgery at AD-A presentation was significantly associated with an increased risk of late reintervention (HR, 2.09; 95% CI, 1.13-3.86; *P* = .019). In 63 patients who had undergone aortic arch surgery, a total of 72 reinterventions were performed, including 48 distal reinterventions (aortic arch or descending aorta) and 24 proximal reinterventions (ascending aorta, aortic root, or aortic valve). For females, prior aortic surgery (HR, 11.9; 95% CI, 1.15-123.5; *P* = .038) and prior cardiac surgery (HR, 11.0; 95% CI, 1.26-96.3; *P* = .032) were significantly associated with late reintervention. No significant associations with late reintervention were observed in males.

## Discussion

Research into cardiovascular health among female patients has been expanding over the last 2 decades.^22^ The current study investigated male–female differences in a large multicenter cohort of AD-A patients. The main findings are that (1) females presented with AD-A at a higher age and more often presented with severe hypotension, nausea, and tamponade; (2) short-term morbidity and mortality were comparable between males and females with AD-A, and sex was not significantly associated with short-term mortality; (3) females had higher unadjusted long-term mortality per patient-year of follow-up compared to males and relatively lower survival compared to the matched general population; (and 4) age, prior myocardial infarction, chronic kidney disease, and prior aortic surgery were independently associated with an increased long-term mortality, whereas female sex was not.

More detailed insight into the presenting symptoms of males and females separately could help optimize prompt diagnosis in both sexes. In turn, faster and more adequate treatment may result in better outcomes. In this large population, (severe) hypotension and cardiac tamponade were observed more often in female patients, and females presented with nausea more frequently compared to males, as was reported previously by Nienaber and colleagues.^10^ Females with acute coronary syndromes also present with nausea and syncope more frequently than males.^23^ Males reported hypoesthesia more often, although this does not seem to indicate more short-term complications, such as transient ischemic attack, cerebrovascular accident, and spinal cord lesions. These differences in symptom patterns suggest that females may experience and report acute events differently than males, indicating that a different approach to diagnosis in males and females may be needed.

When it comes to treatment, the absolute aortic diameter is most commonly used for timing of preventive aortic surgery, as supported by clinical practice guidelines.^6^^,^^16^ Use of the aortic size index (thoracic aortic diameter indexed for BSA) or aortic height index (thoracic aortic diameter indexed for height) has been proposed, but no specific cutoff values for elective aortic surgery are mentioned in the current guidelines.^16^^,^^24^ Our findings showed no significant difference between males and females in absolute aortic diameter before and at the time of AD-A, suggesting that the absolute aortic diameter might be an equally good indicator of disease severity in both males and females. However, our data on aortic diameters at AD-A presentation had 80% missing values. Therefore, our group dedicated a separate study to explore male–female differences in aortic diameters at the time of AD-A presentation.^25^ In this study, females had larger absolute ascending aortic diameters, whereas in the sinus of Valsalva and descending aorta, absolute aortic diameters were comparable in males and females.

Greater short-term mortality in females compared to males with acute aortic dissection has been reported by Smedberg and colleagues^7^ and Nienaber and colleagues.^10^ In contrast, several other studies reported no differences in short-term mortality after AD-A.^26^, ^27^, ^28^, ^29^ In the present large multicenter study, we found no significant male–female differences in short-term mortality and short-term morbidity, even though females more often presented with severe hypotension and tamponade before surgery and were older at the time of presentation. Risk factors for short-term mortality were older age, COPD, descending aortic surgery, and concomitant procedures. Notably, females seem to have a lower observed short-term mortality than the expected mortality, as calculated by the Logistic EuroSCORE, and in our logistic regression analysis female sex was not associated with mortality. These findings suggest that in patients with AD-A, female sex does not contribute to a higher early mortality. Interestingly, in the validation of the more contemporary GERAADA score, male sex was even identified as an independent risk factor for 30-day mortality.^30^ which also supports that female sex does not seem to be an independent risk factor for short-term mortality after AD-A surgery. Given that females with AD-A present at an older age with more comorbidities, studying sex-specific risk factors in future studies reporting on AD-A outcomes could offer valuable insights and improve risk prediction.

Females in this study had lower unadjusted long-term survival and higher overall annual mortality rates than males (8.77% vs 6.51% per patient-year) after surgery for AD-A. Although some studies have reported comparable 5-year^26^^,^^27^^,^^31^, ^32^, ^33^, ^34^ and 10-year^26^^,^^28^^,^^32^^,^^35^ survival for males and females, others, including studies by Gasser and colleagues^36^ and Li and colleagues,^37^ have found impaired long-term survival for females compared to males, even in propensity-matched analyses. Follow-up completeness and heterogeneity in the selection of surgical patients and surgical techniques might explain the contradictory findings across studies. After adjusting for age at surgery and other important patient characteristics, female sex was not associated with increased mortality. Nevertheless, compared to the age- and sex-matched Dutch general population, female patients seemed to have relatively worse survival than male patients. The impaired survival for females is most evident at 5 to 7 years after surgery, a finding consistent with Gasser and colleagues.^36^ There were no significant differences between males and females in the 10-year probability of having any of the cardiovascular events that were registered during follow-up. A more in-depth exploration into causes of death and aortic-related mortality may help clarify the higher mortality rates in females.

In the current study, older age at the time of intervention was a key independent risk factor for long-term mortality in both males and females. Significant sex differences in risk profiles were found; in females, COPD, chronic kidney disease, and a history of thoracic were the main risk factors, whereas in males, prior myocardial infarction was the most important risk factor. Similarly, Suzuki and colleagues^28^ identified distinct independent risk factors for follow-up mortality after AD-A: cardiac tamponade, COPD, and longer operation time in females and older age and preoperative cerebrovascular disease in males. Notably, traditional cardiovascular risk factors, such as myocardial infarction and hypertension, posed a higher mortality risk in females.^38^ It is possible that distinct pathophysiologic processes impact long-term mortality for males and females after AD-A and cardiovascular disease in general.

### Study Limitations

Several limitations apply to this study. First, the retrospective design introduces selection bias and incomplete data, leading to a reduced sample size and limited multivariable modeling. Second, only patients diagnosed with AD-A after reaching the hospital were included, which has been estimated to be roughly 70% of patients with any aortic dissection (Stanford type A and B).^7^ It is unclear whether male–female differences affect the likelihood of reaching the hospital and being diagnosed. Third, some patients were lost to follow-up, potentially biasing results if their loss to follow-up was linked to specific patient characteristics and adverse outcomes (missing not at random). Fourth, differences in surgical techniques among the centers might have led to heterogeneity in the observed outcomes. Finally, survival bias might be present, as the study focused on patients who survived beyond the acute phase of AD-A, mainly for long-term follow-up.

## Conclusions

This study underscores that males and females with AD-A have a distinct clinical profile at presentation, with females presenting at an older age and with more comorbidities. Despite comparable treatment approaches and short-term mortality, unadjusted long-term survival was lower in females, even when compared to the expected survival of the age- and sex-matched general population. Future research should focus on elucidating the underlying mechanisms contributing to the poorer long-term survival in females, in which structured follow-up and collection of causes of death might provide valuable information. Furthermore, the clear differences in risk factors for short- and long-term mortality emphasize the need for implementation of male- and female-specific risk factor stratification and counseling.

## Data Availability Statement

Data will be shared on request to the corresponding author with permission of the disSEXion Study research group.

## Conflict of Interest Statement

The authors report no conflicts of interest.

The *Journal* policy requires editors and reviewers to disclose conflicts of interest and to decline handling or reviewing manuscripts for which they may have a conflict of interest. The editors and reviewers of this article have no conflicts of interest.
