# Supplementary material for: Presentation, management, and clinical outcomes of acute type A dissection: Does sex matter?
Source: JTCVS Open. 2024 Dec 16;24:47–57. doi: 10.1016/j.xjon.2024.12.006 (PMC12039451; doi:10.1016/j.xjon.2024.12.006)
Supplement: Online Data Supplement [file mmc1.docx]

**Supplementary Material**

**Supplemental file 1.** Definitions of variables as defined in case report form.

## **Definitions of variables regarding the patient characteristics**

| Variable | Unit | Definition |
| --- | --- | --- |
| Age at presentation | years | Age (at time of diagnosis) |
| Body surface area | m^2^ | $\sqrt{\frac{height \left( cm \right)x weight (kg)}{3600}}$ |
| Prior aortic surgery |  | Prior aortic surgery reported in patient history |
| Recent myocardial infarction |  | (N)STEMI < 90 days of presentation |
| History of myocardial infarction |  | (N)STEMI > 90 days before presentation |
| History of aortic valve stenosis |  | Known aortic valve stenosis categorised as: grade 1 (minimal), grade 2 (mild), grade 3 (moderate) and grade 4 (severe). |
| History of aortic valve regurgitation? |  | Known aortic valve regurgitation categorised as: grade 1 (minimal), grade 2 (mild), grade 3 (moderate) and grade 4 (severe). |
| Mixed aortic valve disease |  | Known aortic stenosis and regurgitation of at least grade 2 or higher. |
| History of mitral valve regurgitation |  | Known mitral valve regurgitation, categorised as grade 1 (minimal), grade 2 (mild), grade 3 (moderate) and grade 4 (severe). |
| History of hypertension |  | Known hypertension in patient history or medical treatment for hypertension. |
| History of diabetes mellitus |  | Known diabetes mellitus in patient history or medical treatment for diabetes mellitus. |
| History of COPD |  | Any history of Chronic Obstructive Pulmonary Disease that required medical treatment or FEV1<70%. |
| History of CVA/TIA |  | Cerebrovascular accident or transient ischemic attack in patients medical history. |
| History of chronic kidney disease |  | History of chronic kidney disease in medical history. |
| Smoking > 1 pack years |  | Currently smoking or > 1 pack year in past. |
| History of hyperlipidaemia |  | Medical treatment for hyperlipidaemia or in medical history. |
| Known chronic thoracic aortic dissection |  | Known chronic thoracic aortic dissection in medical history. |
| Known thoracic aortic aneurysm prior to presentation |  | Thoracic aortic diameter of ≥ 40 mm. |
| Last measured maximum aortic diameter | mm | Last measured thoracic aortic diameter. |
| Prior dissection or aneurysm in a major artery other than the thoracic aorta |  | Known dissection or aneurysm in a major artery other than the thoracic aorta. |
|  |  |  |
| Bicuspid aortic valve |  | Presence of bicuspid aortic valve, known in patient history or seen in the operation theatre. |
| Presence of connective tissue disease |  | Known in patient history or genetic testing performed after treatment for the aortic dissection. |

##

## **Definitions of variables regarding the clinical presentation**

| Variable | Unit | Definition |
| --- | --- | --- |
| Abrupt onset of symptoms |  | Abrupt onset of symptoms described in patient status. If not reported the variable is registered as unknown. |
| Onset of symptoms during exercise |  | Onset of symptoms during physical activity or sports / exercise. If not reported the variable is registered as unknown. |
| Chest pain |  | As reported in patient file. If not reported the variable is registered as unknown. |
| Back pain |  | As reported in patient file. If not reported the variable is registered as unknown. |
| Abdominal pain |  | As reported in patient file. If not reported the variable is registered as unknown. |
| Radiating pain |  | As reported in patient file. If not reported the variable is registered as unknown. |
| Migrating pain |  | As reported in patient file. If not reported the variable is registered as unknown. |
| Type of pain |  | As reported in patient file. If not reported the variable is registered as unknown. |
| Dyspnoea |  | As reported in patient file. If not reported the variable is registered as unknown. |
| Nausea |  | As reported in patient file. If not reported the variable is registered as unknown. |
| Collapse |  | As reported in patient file. If not reported the variable is registered as no. |
| Hypoesthesia |  | As reported in patient file. If not reported the variable is registered as unknown. |
| Loss of muscle strength |  | As reported in patient file. If not reported the variable is registered as unknown. |
| NYHA classification at presentation |  | New York Heart Association classification. If patient reported dyspnoea in rest at the emergency room, patients were classified NYHA 4. If patients did not report dyspnoea patients were classified NYHA 1. If dyspnoea was not described in patients’ medical records, NYHA classification was unknown. |
| CCS classification at presentation |  | Canadian Cardiovascular Society classification. If patient reported chest pain in rest at the emergency room, patients were classified CCS. If patients did not report chest pain patients were classified CCS 1. If chest pain was not described in patients’ medical records, CCS classification was unknown. |
| Severe hypotension at presentation |  | SBP <90 mmHg or medical treatment for severe hypotension at presentation or before surgery. If not reported the variable is registered as no. |
| Tamponade at or after presentation |  | As reported in patient file, if not reported the variable is registered as no. |
| CVA / TIA at presentation or before surgery |  | As reported in patient file, if not reported the variable is registered as no. |
| Resuscitation needed pre-surgery |  | As reported in patient file, if not reported the variable is registered as no. |
| Difference in right / left arm systolic blood pressure |  | Difference of >20 mmHg between right and left arm blood pressure. |
| Logistic EuroSCORE |  | Calculated on http://www.euroscore.org/calcold.html |
| Duration onset symptoms until diagnosis | hours | If reported in patients’ medical record. Divided into four categories: <6 hours, 6-12 hours, 12-24 hours, and > 24 hours. If there was no information about time to diagnosis, variable was registered as unknown. |
| Blood pressure at presentation | mmHg | Normotensive: Systolic 90-140 mmHg, diastolic 60-90 mmHg Hypotensive: Systolic <90 mmHg or diastolic <60 mmHg Hypertensive: Systolic >140 mmHg or diastolic >90 mmHg |
| Hearth rhythm registered on electrocardiogram. |  | Sinus rhythm (60-100 bpm), sinus bradycardia (<60 bpm), sinus tachycardia (>100 bpm), supraventricular tachycardia, paced rhythm, ventricular tachycardia/fibrillation or other. If not reported the variable is registered as unknown. |
| Any signs of ischemia on ECG |  | As reported by ECG machine or reviewed and reported by a physician. |
| Left ventricular ejection fraction (LVEF) |  | Good (> 55%), reduced (44-55%), moderate (30-45%), poor (<30%) |
| Transthoracic echocardiography (TTE) performed |  | Only if performed after admittance and before surgery. As reported in patient file. If not reported the variable is registered as no. |
| CT-scan performed before surgery |  | Only if performed after admittance and before surgery |
| Transoesophageal echocardiography (TEE) performed |  | Only if TEE was performed before cardiopulmonary bypass. |
| Aortic regurgitation at presentation |  | As reported in patient file, mild, moderate or severe. If not reported the variable is registered as unknown. |
| Maximum aortic diameter | mm | Maximum aortic diameter measured at presentation. |
| Maximum indexed aortic diameter (BSA) | mm/m^2^ | Maximum aortic diameter measured at presentation indexed for BSA. |
| Haemoglobin level | mmol Fe/L | Only if measured after admittance and before surgery. |
| Glomerular filtration rate | ml/min | CKD-EPI eGFR, maximum 1 year before admittance |
| Creatinine | micromole/L | Only if measured after admittance and before surgery |
| Troponin T level | microgram/mL | Highest measured level after admittance and before surgery |
| CK level | U/L | Only if measured after admittance and before surgery |
| CKMB level | microgram/L | Highest measured level after admittance and before surgery |
| ASAT level | U/L | Only if measured after admittance and before surgery |
| ALAT level | U/L | Only if measured after admittance and before surgery |
| LDH level | U/L | Only if measured after admittance and before surgery. If measured before 04/10/2010, LDH was converted from an old LD measurement using the following formula: “LDH=0,5553 x LDH-old – 5,8296” |
| C-reactive protein level | mg/L | Highest measured level after admittance and before surgery |
| D-dimer level | microgram/mL | Highest measured level after admittance and before surgery |
| Leukocyte level | *10^9/L | Only if measured after admittance and before surgery |
| Lactate level | mmol/L | Only if measured after admittance and before surgery |
| Surgery performed or attempted |  | If surgery was performed or attempted, then yes. If surgery was not performed or attempted, then no. |

**Definitions of variables regarding the postoperative hospital stay**

| Variable | Unit | Definition |
| --- | --- | --- |
| Short-term mortality |  | Death within 30 days or before hospital discharge. Pre-surgery, in operating theatre or post-surgery. |
| Cause of in hospital death |  | Cardiac, neurologic, organ failure, aortic rupture, bleeding, sepsis or ‘other’ cause. |
| Number of days the patient was admitted | days | Total time in days from admission until discharge |
| Number of days in intensive care unit (ICU) after surgery | days | Number of days patient was on ICU after surgery. |
| Reoperation needed |  | Reoperation during admission or within 30 days. |
| Indication for reoperation |  | Bleeding event, tamponade, mediastinitis, mechanical sternum dehiscence, non-structural valve dysfunction, non -operated valve dysfunction or ‘other’ indication. |
| Diagnosis of any infection after surgery? |  | During admission or within 30 days after surgery. |
| Diagnosis of sepsis after surgery? |  | During admission or within 30 days after surgery. |
| Myocardial infarction or ischemia after surgery |  | During admission or within 30 days after surgery. |
| CVA diagnosed after surgery? |  | During admission or within 30 days after surgery. |
| TIA after Surgery? |  | During admission or within 30 days after surgery. |
| Spinal cord lesion after surgery |  | During admission or within 30 days after surgery. |
| Lowest eGFR measured during admittance after surgery? |  | Lowest CKD-EPI eGFR during admission or within 30 days after surgery. |

## **Definitions of variables regarding the follow-up data**

| Long-term follow-up |  | Long-term follow-up is defined as the follow-up period starting after hospital stay and > 30 days after diagnosis until the mortality check |
| --- | --- | --- |
| Patient years |  | The value of the patients years is determined as the number of patients multiplied by the mean follow-up time |
| Mortality |  | All-cause mortality |
| Reintervention |  | An intervention (percutaneous or surgical) on the thoracic aorta or side branches, related to the aortic dissection  The definition of a late reintervention is in accordance with Akins et al. (1) |
| Reintervention - proximal |  | Ascending aorta, aortic root or aortic valve |
| Reintervention – distal |  | Aortic arch or descending aorta |
| Thoracic aortic aneurysm |  | Thoracic aortic aneurysm requiring percutaneous or surgical intervention |
| False aneurysm |  | False aneurysm/seam leakage/seam aneurysm requiring percutaneous or surgical intervention |
| Abdominal aortic aneurysm |  | Abdominal aortic aneurysm or rupture requiring percutaneous or surgical intervention |
| Thoracic aortic dissection |  | History of a dissection in the long-term follow-up until the date of the signed informed consent.  The definition of a dissection is in accordance with Erbel et al. (2) |
| Myocardial infarction |  | Myocardial infarction diagnosis based on clinical report |
| CVA |  | CVA diagnosis based on clinical report. The definition of a CVA is in accordance with Akins et al. (1). |
| TIA |  | TIA diagnosis based on clinical report. The definition of a TIA is in accordance with Akins et al. (2008) (1). Including amaurosis fugax. |
| Bleeding |  | The definition of a bleeding event is in accordance with Akins et al. (1) |
| Endocarditis |  | Endocarditis event as defined by Akins et al. (1). When patients received antibiotic treatment or surgical intervention for endocarditis, it was considered as endocarditis event. |
| Operative valve dysfunction |  | Structural or non-structural valve deterioration as defined according to Akins et al (1). Only for patients who had aortic valve replacement at ATAAD surgery. Considered as event if catheter procedure or open surgical procedure was performed. |
| NYHA |  | New York Heart Association score as filled in by the participant in the questionnaire and otherwise scored based on clinical report |
| Pacemaker / ICD implantation |  | Pacemaker or ICD implantation |

CVA = cerebrovascular accident; TIA = transient ischemic attack; NYHA = New York Heart Association

1. Akins CW, Miller DC, Turina MI, Kouchoukos NT, Blackstone EH, Grunkemeier GL, et al. Guidelines for reporting mortality and morbidity after cardiac valve interventions. Ann Thorac Surg. 2008;85(4):1490-5.

2. Erbel R, Aboyans V, Boileau C, Bossone E, Bartolomeo RD, Eggebrecht H, et al. 2014 ESC Guidelines on the diagnosis and treatment of aortic diseases: Document covering acute and chronic aortic diseases of the thoracic and abdominal aorta of the adult. The Task Force for the Diagnosis and Treatment of Aortic Diseases of the European Society of Cardiology (ESC). Eur Heart J. 2014;35(41):2873-926.

**Supplemental file 2**. Male-female differences in clinical presentation

|  | All  N=889 | Male patients  N= 558 | Female patients  N= 331 | P value | Missing |
| --- | --- | --- | --- | --- | --- |
| Any symptoms | 689 (97.2) | 429 (97.7) | 256 (96.2) | 0.360 | 20.7 |
| Abrupt onset | 446 (98.5) | 283 (98.3) | 163 (98.8) | 1.000^e^ | 49.0 |
| During exercise | 78 (27.9) | 55 (31.1) | 23 (22.3) | 0.151 | 68.5 |
| Type of pain  No pain  Tearing  Sharp  Oppresive | 11 (4.1)  26 (9.7)  68 (25.5)  162 (60.7) | 7 (4.1)  21 (12.4)  43 (25.3)  99 (58.2) | 4 (4.1)  5 (5.2)  25 (25.8)  63 (64.9) | 0.290 | 70.0 |
| Radiating pain | 266 (74.9) | 168 (76.4) | 98 (72.6) | 0.503 | 60.1 |
| Migrating pain | 7 (47.5) | 49 (49.5) | 27 (44.3) | 0.631 | 82.0 |
| NYHA class  I  II  III  IV | 135 (61.1)  7 (3.2)  4 (1.2)  74 (33.6) | 87 (61.7)  5 (3.5)  3 (2.1)  46 (32.6) | 48 (60.0)  2 (2.5)  1 (1.2)  28 (35.4) | 0.974 ^e^ | 75.3 |
|  |  |  |  |  |  |
| BP difference | 109 (38.5) | 77 (42.3) | 32 (31.7) | 0.103 | 68.2 |
| BP  Hypotensive  Normotensive  Hypertensive | 193 (30.1)  326 (50.9)  122 (19.0) | 97 (24.6)  215 (54.6)  83 (20.8) | 96 (38.9)  111 (44.9)  40 (16.2) | <0.001  0.017  0.150 | 27.9 |
| Severe hypotension | 171 (23.7) | 86 (19.4) | 85 (30.7) | <0.001** | 18.9 |
| Tamponade  Pericardiocentesis | 149 (21.0)  6 (0.9) | 78 (18.0)  5 (1.1) | 71 (25.6)  1 (0.4) | 0.019*  0.417 ^e^ | 20.0  21.4 |
| CVA | 49 (9.3) | 30 (9.2) | 29 (9.3) | 0.774 | 40.0 |
| Resuscitation | 60 (8.3) | 30 (6.6) | 30 (11.0) | 0.052 | 18.2 |
| Renal failure | 26 (4.5) | 15 (4.2) | 11 (5.0) | 0.806 | 34.8 |
| Logistic EuroScore | 22.4 [12.2; 37.7] | 18.0 [9.3; 30.3] | 30.3 [20.0; 47.4] | <0.001** | 20.2 |
| Time from symptom onset to diagnosis  <6h  6-11.59h  12-48h  >48h | 366 (64.1)  55 (9.6)  73 (12.8)  77 (13.5) | 234 (66.3)  27 (7.6)  44 (12.5)  48 (13.6) | 132 (60.6)  28 (12.8)  29 (13.3)  29 (13.3) | 0.208 | 35.8 |

Continuous data are presented as mean ± standard deviation or as median (interquartile range) as appropriate. Categorical data are presented as absolute and percentage.

NYHA class= New York Heart Association class; CCS class= Canadian Cardiovascular Society class; BP= Blood Pressure; CVA= Cerebrovascular Accident including Transient Ichemic Attack.

* Significant at the 0.05 level

** Significant at the 0.01 level

^e^ Fishers exact test

**Figure 2a.** Male-female differences in symptoms at AD-A presentation


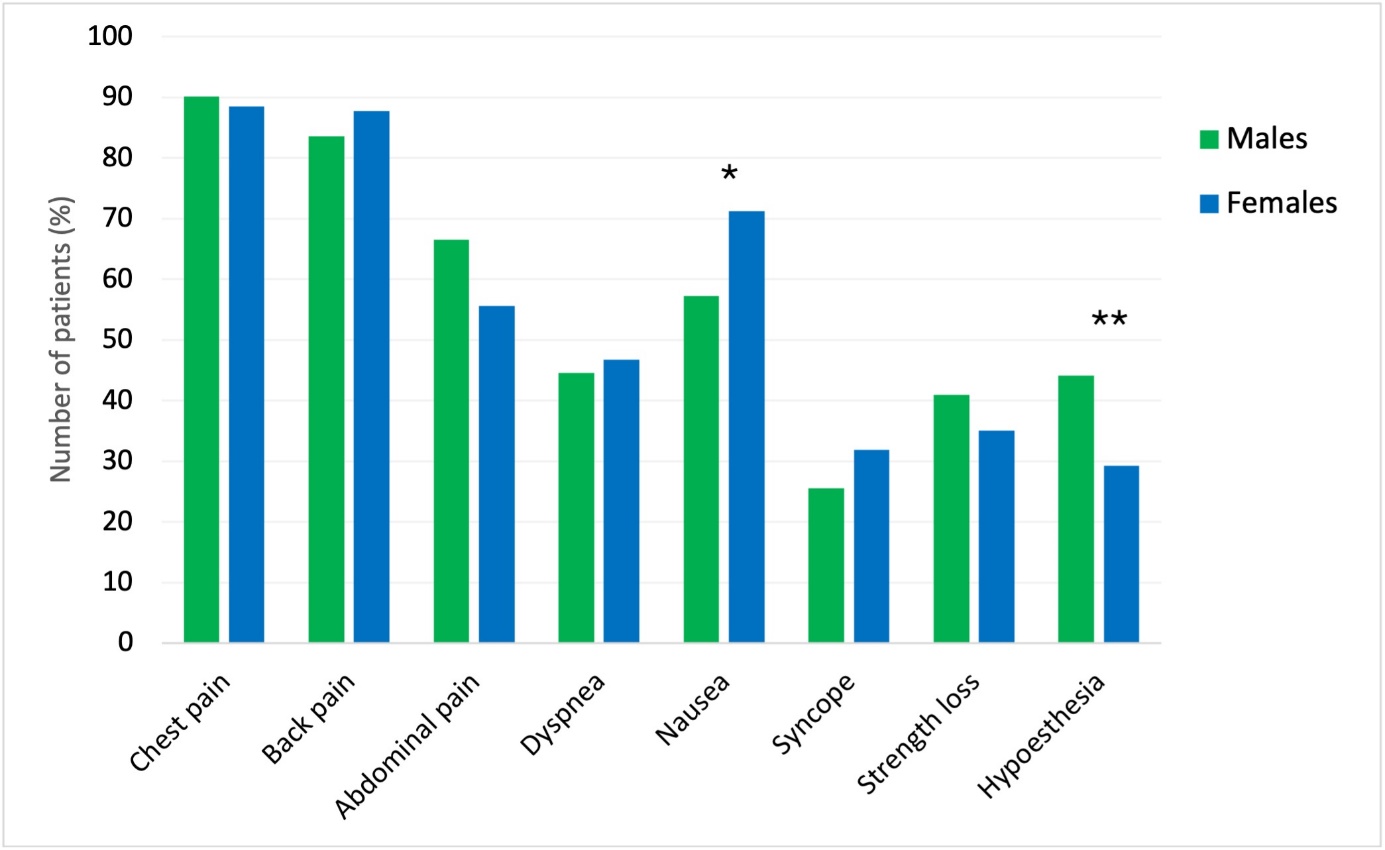


**Supplemental file 3a.** Diagnostic imaging results

|  | | All  N= 889 | | Male patients  N= 558 | | Female patients  N= 331 | | P value | | Missing | |
| --- | --- | --- | --- | --- | --- | --- | --- | --- | --- | --- | --- |
| DeBakey classification  I  II | | 690 (87.7)  97 (12.3) | | 446 (88.8)  56 (11.2) | | 244 (85.6)  41 (14.4) | | 0.255 | | 11.5 | |
| Location of intimal tear  Ascending aorta  Aortic arch  Descending aorta | | 535 (75.5)  158 (22.2)  16 (2.3) | | 346 (75.4)  99 (21.6)  14 (3.1) | | 189 (75.6)  59 (23.6)  2 (0.8) | | 0.131^e^ | | 20.4 | |
| Rhythm registered on ECG | |  | |  | |  | | 0.375^e^ | | 36.3 | |
| Sinus rhythm (60-100 bpm) | | 375 (66.3) | | 220 (64.0) | | 155 (69.8) | |  | |  | |
| Sinus tachycardia (>100 bpm) | | 21 (3.7) | | 16 (4.7) | | 5 (2.3) | |  | |  | |
| Sinus bradycardia (<60 bpm) | | 119 (21.0) | | 78 (22.7) | | 41 (18.5) | |  | |  | |
| Supraventricular tachycardia  Ventricular tachycardia  Other | | 33 (5.8)  1 (0.2)  15 (2.7) | | 21 (6.1)  0 (0.0)  8 (2.3) | | 12 (5.4)  1 (0.5)  7 (3.2) | |  | |  | |
| Any signs of ischemia on ECG | | 140 (27.1) | | 92 (29.1) | | 48 (23.9) | | 0.223 | | 41.8 | |
| Measurement of LVEF - % | |  | |  | |  | | 0.400^e^ | | 49.7 | |
| Normal (>55%) | | 385 (86.1) | | 234 (85.4) | | 151 (87.3) | |  | |  | |
| Reduced (45-55%) | | 38 (8.5) | | 26 (9.5) | | 12 (6.9) | |  | |  | |
| Moderate (30-45%) | | 18 (4.0) | | 12 (4.4) | | 6 (3.5) | |  | |  | |
| Poor (<30%) | | 6 (1.3) | | 2 (0.7) | | 4 (2.3) | |  | |  | |
| Severity of aortic regurgitation | |  | |  | |  | | 0.333 | | 50.6 | |
| Mild | | 78 (17.8) | | 53 (19.3) | | 25 (15.2) | |  | |  | |
| Moderate | | 73 (16.6) | | 43 (15.6) | | 30 (18.3) | |  | |  | |
| Severe | | 77 (17.5) | | 53 (19.3) | | 24 (14.6) | |  | |  | |
| Maximum aortic diameter - mm | | 53.0 [48.0-59.5] | | 53.0 [49.0-60.0] | | 53.0 [47.0-59.0] | | 0.600 | | 80.8 | |
| Maximum aortic diameter indexed for BSA - mm/m^2^ | | 26.7 [24.5-31.6] | | 26.1 [23.6-29.0] | | 28.5 [25.5-32.4] | | 0.001** | | 84.4 | |
| Aortic Rupture | | 39 (9.4) | | 24 (9.1) | | 15 (9.9) | | 0.914 | | 53.3 | |
| Pleural effusion | | 42 (13.3) | | 24 (12.4) | | 18 (14.8) | | 0.662 | | 64.5 | |
| Pericardial effusion | | 354 (53.2) | | 218 (53.2) | | 136 (53.3) | | 1.000 | | 25.2 | |

Continuous data are presented as mean ± standard deviation or as median (interquartile range) as appropriate. Categorical data are presented as absolute and percentage.

ECG= Electrocardiography; LVEF= Left Ventricular Ejection Fraction; BSA= Body Surface Area.

** Significant at the 0.01 level

^e^ Fishers exact test

**Supplemental file 3b.** Comparison of abnormal laboratory results in males and females presenting with AD-A.

**

Data are presented as percentage of males and females with abnormal results.

AD-A: Acute Stanford type A thoracic aortic dissection; GFR= Glomerular Filtration Rate; CK= Creatinin Kinase; CKMB= Creatinin Kinase Myocardial Band; Hs TropT= High Sensitive Troponin T; ASAT= Aspartate Transaminase; ALAT= Alanine Transaminase; LD= Lactate Dehydrogenase.

** Significant at the 0.01 level

**Supplemental file 4a.** Univariable logistic regression analysis on factors associated with mortality short term mortality excluding nonsurgical patients.

|  | | **Total n=847** |  | **Males**  **n= 532** |  | **Females**  **n= 315** |  |
| --- | --- | --- | --- | --- | --- | --- | --- |
| **Variable** | | **OR (95%CI)** | **P value** | **OR (95%CI)** | **P value** | **OR (95%CI)** | **P value** |
| ***Baseline*** | |  |  |  |  |  |  |
| Age | | 1.03 (1.02; 1.05) | <0.001** | 1.03 (1.01; 1.05) | <0.001** | 1.04 (1.01; 1.07) | 0.004** |
| Sex | | 1.19 (0.85; 1.66) | 0.318 | - | - | - | - |
| History of hypertension | | 1.17 (0.82; 1.67) | 0.379 | 0.96 (0.60; 1.50) | 0.844 | 1.58 (0.89; 2.89) | 0.128 |
| History of hyperlipidaemia | | 1.05 (0.58; 1.79) | 0.868 | 0.93 (0.39; 1.94) | 0.848 | 1.16 (0.50; 2.48) | 0.718 |
| COPD | | 2.93 (1.68; 5.00) | <0.001** | 3.50 (1.64; 7.22) | <0.001** | 2.29 (0.98; 5.08) | 0.046* |
| Diabetes | | 1.82 (0.74; 4.08) | 0.156 | 1.54 (0.40; 4.54) | 0.455 | 2.19 (0.57; 7.20) | 0.213 |
| Prior CVA | | 1.43 (0.66; 2.88) | 0.333 | 1.81 (0.64; 4.51) | 0.223 | 1.04 (0.29; 2.95) | 0.948 |
| Prior MI | | 1.30 (0.58; 2.68) | 0.494 | 0.93 (0.27; 2.52) | 0.901 | 1.93 (0.59; 5.54) | 0.238 |
| Chronic kidney disease | | 2.25 (0.90; 5.17) | 0.064 | 1.24 (0.19; 5.06) | 0.786 | 3.00 (0.97; 8.66) | 0.045* |
| Prior aortic surgery | | 1.25 (0.45; 2.95) | 0.638 | 1.66 (0.53; 4.40) | 0.341 | 0.58 (0.03; 3.32) | 0.609 |
| Prior cardiac surgery | | 1.12 (0.89; 1.37) | 0.310 | 1.23 (0.84; 1.43) | 0.380 | 1.12 (0.71; 1.62) | 0.549 |
| Chronic dissection | | 0.78 (0.04; 4.61) | 0.819 | 2.53 (0.12; 15.67) | 0.451 | 0.00 (-; 1.21) | 0.984 |
| Known aortic aneurysm | | 1.43 (0.77; 2.52) | 0.232 | 1.18 (0.46; 2.64) | 0.703 | 1.68 (0.70; 3.70) | 0.218 |
| BAV | | 1.16 (0.33; 3.19) | 0.795 | 0.83 (0.13; 3.10) | 0.813 | 2.14 (0.29; 11.23) | 0.388 |
| ***Presentation*** |  |  |  |  |  |  |  |
| DeBakey classification | | 1.06 (0.60; 1.79) | 0.841 | 0.70 (0.28; 1.50) | 0.391 | 1.57 (0.71; 3.28) | 0.246 |
| ***Treatment and surgery*** | |  |  |  |  |  |  |
| Aortic valve surgery | | 1.08 (1.01; 1.15) | 0.642 | 1.05 (0.96; 1.14) | 0.799 | 1.13 (1.02; 1.25) | 0.652 |
| Hemi-arch replacement | | 0.66 (0.46; 0.94) | 0.021* | 0.86 (0.54; 1.34) | 0.497 | 0.44 (0.24; 0.78) | 0.006** |
| (Partial) arch replacement | | 1.30 (0.82; 2.03) | 0.251 | 1.25 (0.69; 2.17) | 0.452 | 1.44 (0.66; 2.97) | 0.335 |
| Descending aortic surgery | | 3.24 (1.26; 9.25) | 0.015* | 3.64 (1.15; 13.77) | 0.014* | 2.61 (0.43; 18.55) | 0.548 |
| Concomitant procedures | | 3.54 (2.08; 5.94) | <0.001** | 4.20 (2.12; 8.20) | <0.001** | 2.71 (1.14; 6.16) | 0.018* |
| ***Post-surgery*** | |  |  |  |  |  |  |
| Reoperation | | 1.39 (0.96; 2.02) | 0.081 | 1.49 (0.92; 2.37) | 0.099 | 1.30 (0.69; 2.38) | 0.408 |
| Device implantation | | 1.96 (0.43; 6.88) | 0.324 | 2.84 (0.39; 14.78) | 0.233 | 1.15 (0.06; 7.97) | 0.900 |
| MI | | 6.31 (2.23; 18.30) | <0.001** | 3.48 (0.70; 14.46) | 0.093 | 12.79 (2.68; 91.06) | 0.003** |
| Bleeding | | 1.84 (1.24; 2.71) | 0.002** | 1.86 (1.13; 3.04) | 0.014* | 1.89 (0.97; 3.57) | 0.055 |
| CVA | | 3.23 (1.99; 5.16) | <0.001** | 2.61 (1.35; 4.87) | 0.003** | 4.23 (2.03; 8.68) | <0.001** |
| Infection | | 0.79 (0.53; 1.18) | 0.262 | 0.89 (0.52; 1.47) | 0.653 | 0.66 (0.34; 1.24) | 0.214 |
| Short-term mortality= Intra– or postoperative death < 30 days or within hospital stay  HR= Hazard ratio, 95% CI= 95% Confidence Interval; COPD= Chronic Obstructive Pulmonary Disease; CVA= Cerebrovascular Accident; MI= Myocardial Infarction, BAV= Bicuspid Aortic Valve.  * Significant at the 0.05 level  ** Significant at the 0.01 level | | | | | | | |

**Supplemental file 4b.** Multivariable logistic regression analysis for short mortality excluding preoperative deaths with adjustment for study centre

|  | **Total n=847** |  |
| --- | --- | --- |
| **Variable** | **OR (95%CI)** | **P value** |
| ***Baseline*** |  |  |
| Age | 1.04 (1.02; 1.06) | <0.001 |
| COPD | 2.89 (1.57; 5.31) | <0.001 |
| Descending aortic surgery | 6.76 (1.69; 27.05) | 0.007 |
| Concomitant procedures | 4.25 (2.44; 7.41) | <0.001 |

**Supplemental file 5**

Observed versus expected 30-day mortality according to the Logistic Euroscore.

**
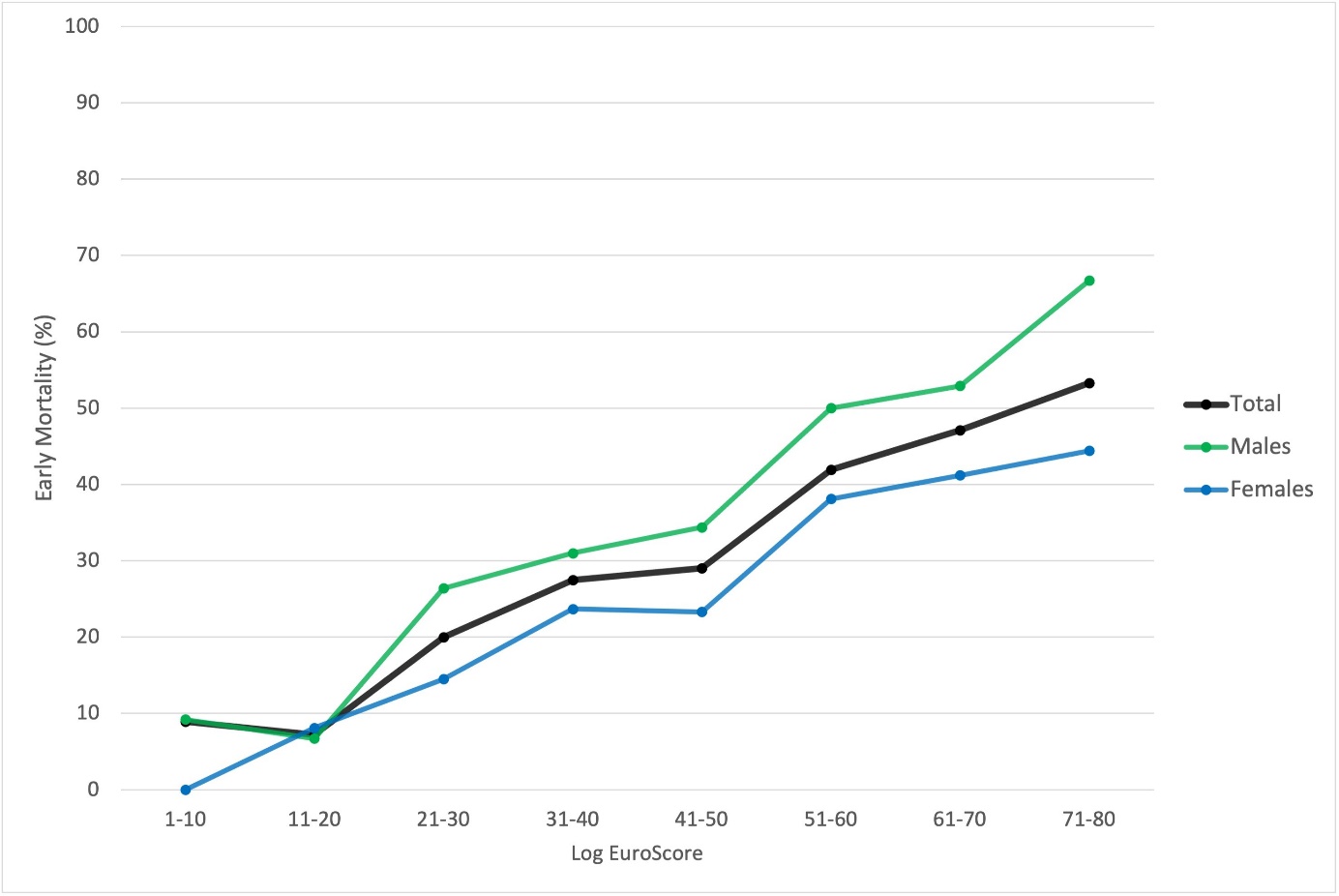
**

**Supplemental file 6.** Flowchart of patient selection

*Long term follow-up included cardiovascular events as mentioned in the questionnaire, and/or as checked in the patient files of the treating hospital


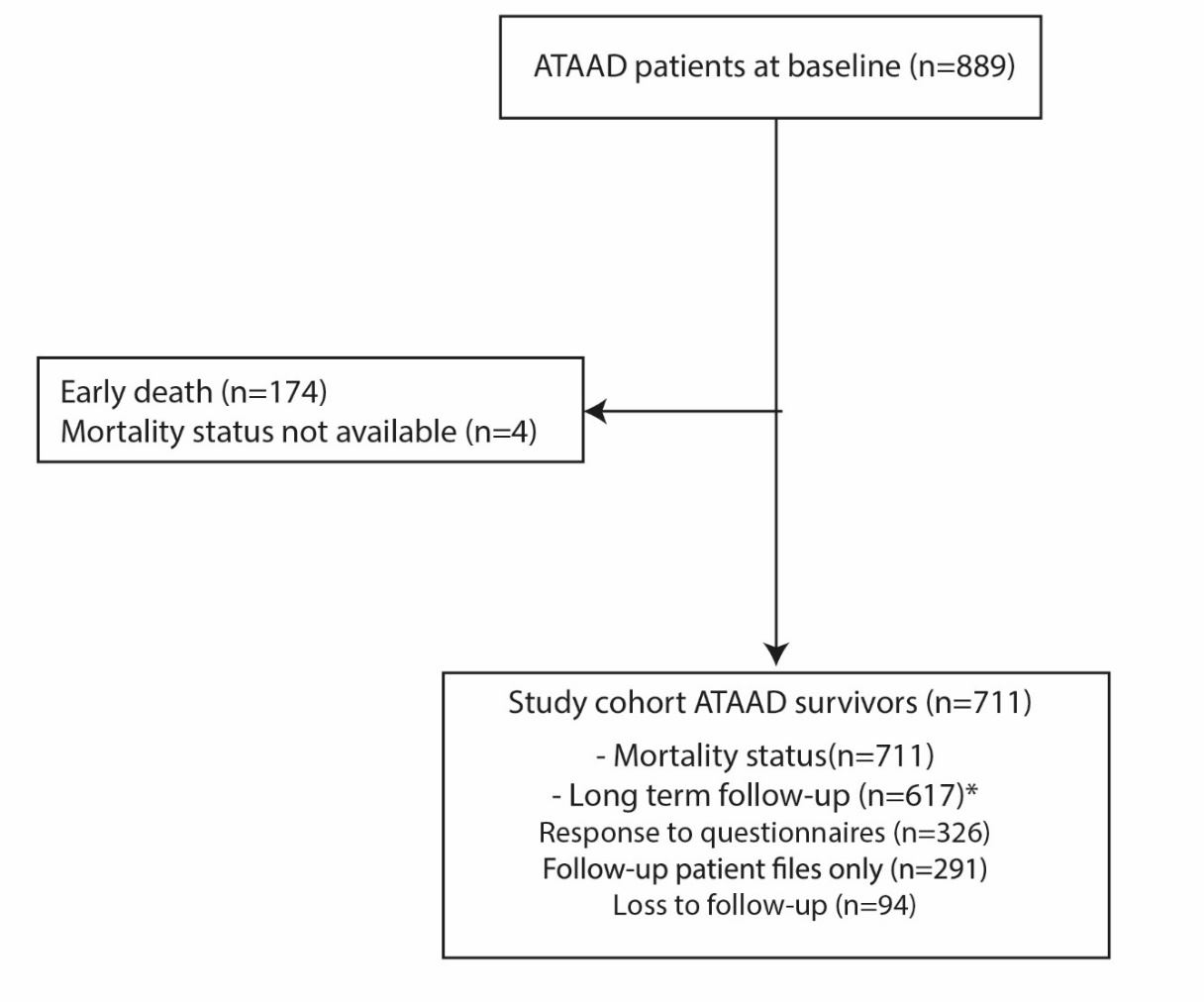


**Supplemental file 7.** Patient and treatment characteristics at presentation of AD-A survivors

|  | All patients (n=711) | Females  (n=260) | Males  (n=451) | P-value | Missing % |
| --- | --- | --- | --- | --- | --- |
| *Patient demographics* |  |  |  |  |  |
| Age (median [IQR]) – years | 62.0  [54.0-70.0] | 65.5  [58.0-74.0] | 60.0  [53.0-68.0] | <0.001 | 0.3 |
| BSA (mean ± SD) – m^2^ | 1.99 ± 0.22 | 1.79 ± 0.16 | 2.10 ± 0.17 | <0.001 | 26.3 |
| History of hypertension (%) | 342 (50.3) | 144 (57.4) | 198 (46.2) | 0.006 | 4.4 |
| Hyperlipidaemia (%) | 75 (10.8) | 32 (12.6) | 43 (9.7) | 0.294 | 2.1 |
| Diabetes mellitus (%) | 14 (2.0) | 8 (3.1) | 6 (1.4) | 0.189 | 1.4 |
| COPD (%) | 41 (5.8) | 20 (7.7) | 21 (4.7) | 0.137 | 0.7 |
| Current or past smoking > 1 pack years | 228 (72.6) | 76 (67.3) | 152 (75.6) | 0.143 | 55.8 |
| History of CVA/TIA (%) | 33 (4.7) | 16 (6.2) | 17 (3.8) | 0.206 | 1.3 |
| History of MI (%) | 32 (4.6) | 11 (4.2) | 21 (4.7) | 0.909 | 1.3 |
| Chronic kidney disease (%) | 17 (2.4) | 9 (3.5) | 8 (1.8) | 0.244 | 1.1 |
| Prior TAA (%) | 53 (7.6) | 24 (9.3) | 29 (6.6) | 0.238 | 1.8 |
| Prior aortic surgery (%) | 21 (3.0) | 7 (2.7) | 14 (3.2) | 0.916 | 1.4 |
| Prior cardiac surgery (%) | 32 (4.5) | 7 (2.7) | 25 (5.6) | 0.109 | 1.0 |
| Bicuspid aortic valve (%) | 17 (2.7) | 4 (1.7) | 13 (3.3) | 0.313^i^ | 10.8 |
| Known connective tissue disease (%)*  No; no genetic testing performed  No; genetic testing performed but not found | 28 (11.6)  139 (57.7)  74 (30.7) | 11 (13.6)  44 (54.3)  26 (32.1) | 17 (10.6)  95 (59.4)  17 (10.6) | 0.698 | 66.1 |
| *Surgical procedure*** |  |  |  |  |  |
| Aortic valve surgery  Ascending aortic surgery  Aortic arch surgery  Descending aortic surgery | 416 (60.6)  695 (98.3)  477 (69.0)  5 (0.7) | 147 (58.6)  252 (97.7)  309 (70.5)  2 (0.8) | 269 (61.7)  443 (98.7)  168 (66.4)  3 (0.7) | 0.467  0.498  0.294  1.000^i^ | 3.4  0.6  2.8  0.6 |

Normally distributed continuous variables are expressed as mean ± SD, skewed continuous variables are expressed as median and 25^th^-75^th^ percentile, and categorical values are expressed as percentages. For follow-up time, the 25^th^-75^th^ percentile as well as the range are reported. P-values < 0.05 are depicted in bold. *Known connective tissue disease at TAAD presentation: Marfan syndrome (n=6), Loeys-Dietz syndrome (n=3), Ehlers-Danlos syndrome type IV (n=1), Turner syndrome (n=2), ACTA2 mutation (n=6), other (n=7). **All patients received surgical treatment. Patients are overlapping in each surgical group.

^i^ = Fisher’s exact test. IQR = interquartile range; BSA = body surface area; SD = standard deviation; COPD = chronic obstructive pulmonary disease; CVA = cerebrovascular accident; TIA = transient ischemic attack; MI = myocardial infarction; TAA = thoracic aortic aneurysm

**
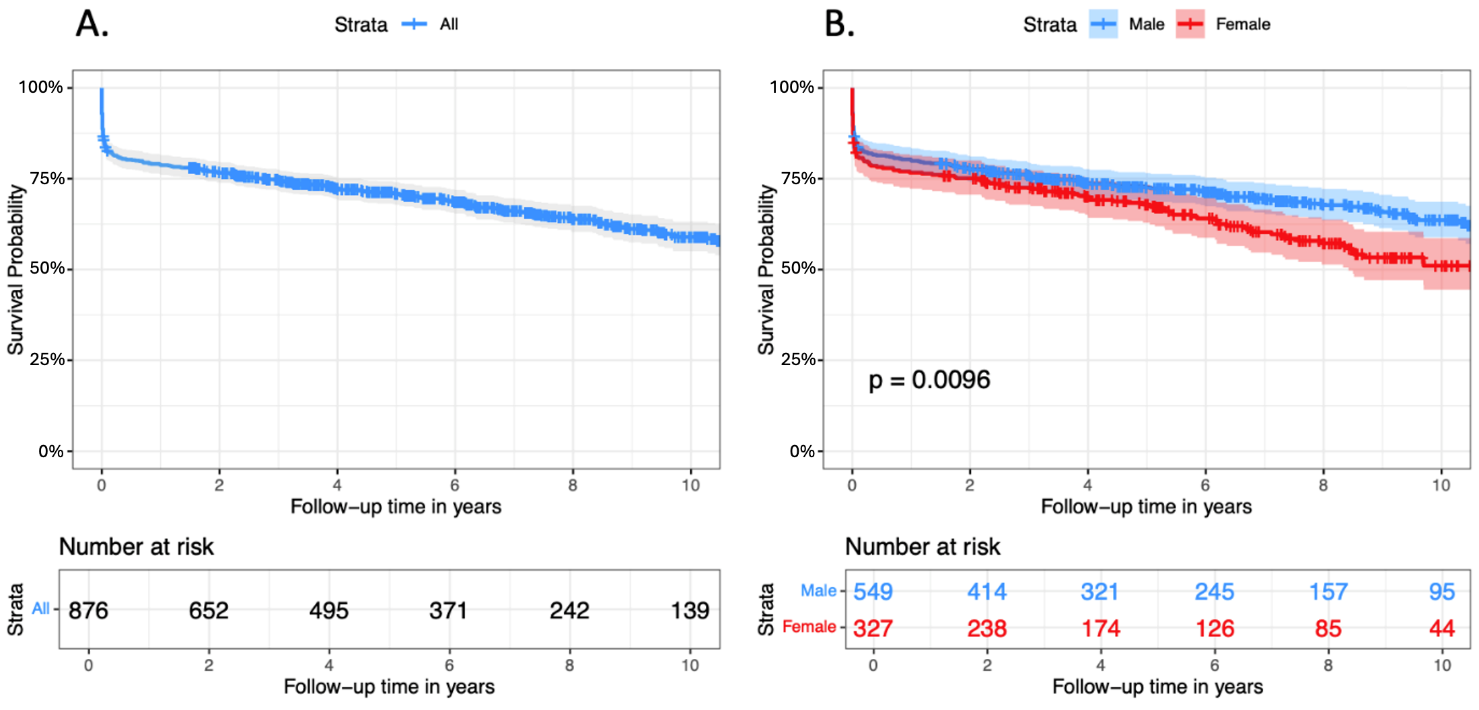
Supplemental figure 8.** Kaplan-Meier estimates for surgical mortality excluding preoperative deaths for the total cohort presented in panel A, and stratified by sex presented in panel B.

**Supplemental file 9.** Cardiovascular events depicted as 10 year event-free probabilities for the total cohort and stratified for males and females.

|  | Total population |  | Females |  | Males |  |  |  |
| --- | --- | --- | --- | --- | --- | --- | --- | --- |
|  | Total number of patients with event | 10 year event-free probability (95%CI) | Total number of patients with event | 10 year event-free probability (95%CI) | Total number of patients with event | 10 year event-free probability (95%CI) | HR (95% CI)* | P-value |
| Overall mortality | 317 | 58.1 (54.2-62.3) | 137 | 50.1 (43.6-57.6) | 180 | 65.0 (60.6-69.7) | 1.34 (1.08-1.69) | 0.009 |
| Long term mortality | 143 | 72.3 (67.9-76.8) | 55 | 63.8 (56.1-72.4) | 77 | 77.1 (72.1-82.4) | 1.60 (1.15-2.22) | 0.006 |
| Long term reintervention*   - Proximal - Distal | 81  34  62 | 81.6 (77.5-85.9)  91.8 (89.0-94.8)  86.6 (83.0-90.4) | 26  13  21 | 80.5 (72.8-89.0)  90.0 (84.3-96.0)  85.4 (78.6-92.9) | 55  21  41 | 82.0 (77.2-87.1)  92.8 (89.7-96.1)  87.1 (82.9-91.4) | 0.84 (0.52-1.34)  1.11 (0.56-2.23)  0.93 (0.55-1.58) | 0.457  0.759  0.789 |
| Thoracic aortic aneurysm | 58 | 84.9 (80.6-89.4) | 17 | 83.0 (74.1-93.0) | 41 | 85.5 (80.9-90.5) | 0.73 (0.41-1.28) | 0.268 |
| False aneurysm | 22 | 93.6 (90.7-96.7) | 11 | 90.8 (85.1-96.8) | 11 | 95.1 (91.9-98.5) | 1.93 (0.83-4.48) | 0.125 |
| Abdominal aortic aneurysm | 13 | 96.7 (94.6-98.8) | 1 | 99.2 (97.6-100.0) | 12 | 95.4 (92.4-98.4) | 0.15 (0.02-1.14) | 0.066 |
| Thoracic aortic dissection | 5 | 98.9 (97.8-100.0) | 2 | 99.5 (98.5-100.0) | 3 | 98.7 (97.0-100.0) | 1.31 (0.22-7.86) | 0.766 |
| Myocardial infarction | 5 | 99.1 (98.4-99.9) | 0 | 100.0 | 5 | 98.6 (97.5-99.8) | - | 0.999 |
| CVA | 29 | 92.4 (89.1-95.8) | 6 | 94.9 (89.8-1.00) | 23 | 91.1 (86.9-95.5) | 0.51 (0.21-1.26) | 0.144 |
| TIA | 31 | 93.6 (91.2-96.1) | 8 | 95.1 (91.3-99.2) | 23 | 92.8 (89.7-95.9) | 0.65 (0.29-1.46) | 0.298 |
| Bleeding | 5 | 99.2 (98.5-100.0) | 0 | 100.0 | 5 | 98.8 (97.6-100.0) | - | 0.999 |
| Endocarditis | 15 | 96.8 (95.0-98.6) | 4 | 97.6 (95.3-100.0) | 11 | 96.4 (93.9-98.9) | 0.68 (0.22-2.15) | 0.515 |
| Operative valve dysfunction^┼^ | 7 | 97.2 (95.0-99.5) | 2 | 96.6 (91.8-100.0) | 5 | 97.5 (95.3-99.7) | 0.69 (0.13-3.55) | 0.654 |

The number of patients with an event and the 10-year event free probability with corresponding 95% confidence interval (CI) are depicted for the total cohort and males and females separately. Hazard ratios with a stratified baseline hazard for study centre and corresponding 95% CI were calculated comparing females to males. *Considered proximal if the ascending aorta, aortic root or aortic valve were involved and distal if the aortic arch or descending aorta were involved in the procedure. ^┼^Only for patients who underwent aortic valve replacement or repair at ATAAD surgery.

CI = confidence interval; CVA = cerebrovascular accident; TIA = transient ischemic attack

**Supplemental file 10.** Specifics of remaining cardiovascular outcomes during follow-up.

|  | Total population | Females | Males | P-value | Missing % |
| --- | --- | --- | --- | --- | --- |
| Late death – cause   - Aortic rupture/vascular - Cardiac - Neurological - Reoperation - Malignancy - Other | 5 (25.0)  4 (20.0)  2 (10.0)  5 (25.0)  2 (10.0)  2 (10.0) | 2 (40.0)  1 (20.0)  0 (0.0)  2 (40.0)  0 (0.0)  0 (0.0) | 3 (20.0)  3 (20.0)  2 (13.3)  3 (20.0)  2 (13.3)  2 (13.3) | 1.000^i^ | 97.2 |
| NYHA class   - NYHA I - NYHA II - NYHA III - NYHA IV | 219 (55.6)  114 (28.9)  54 (13.7)  7 (1.8) | 69 (50.4)  34 (24.8)  29 (21.2)  5 (3.6) | 150 (58.4)  80 (31.1)  25 (9.7)  2 (0.8) | 0.002^i^ | 44.2 |
| Pacemaker/ICD implanted   - No - PM - ICD | 583 (95.6)  21 (3.4)  6 (1.0) | 211 (96.3)  5 (2.3)  3 (1.4) | 372 (95.1)  16 (4.1)  3 (0.8) | 0.417^i^ | 14.2 |
| Indication PM/ICD   - AV-block - Sick-sinus syndrome - Bradycardia - Other | 9 (52.9)  1 (5.9)  2 (11.8)  5 (29.4) | 2 (50.0)  0 (0.0)  1 (25.0)  1 (25.0) | 7 (53.8)  1 (7.7)  1 (7.7)  4 (30.8) | 0.824^i^ | 97.6 |

NYHA = New York Heart Association; PM = pacemaker; ICD = implantable cardioverter defibrillator; AV-block = atrioventricular block

Valve dysfunction only for patients with initial valve repair/replacement. HR with stratified baseline hazard for study centre.

**Supplemental file 11.** Long term reintervention for males and females depicted as A) Kaplan-Meier estimates for freedom from reintervention and B) cumulative incidence of reintervention when accounting for death as competing risk. The Gray’s test comparing males with females was not significant (p=0.40).

1.
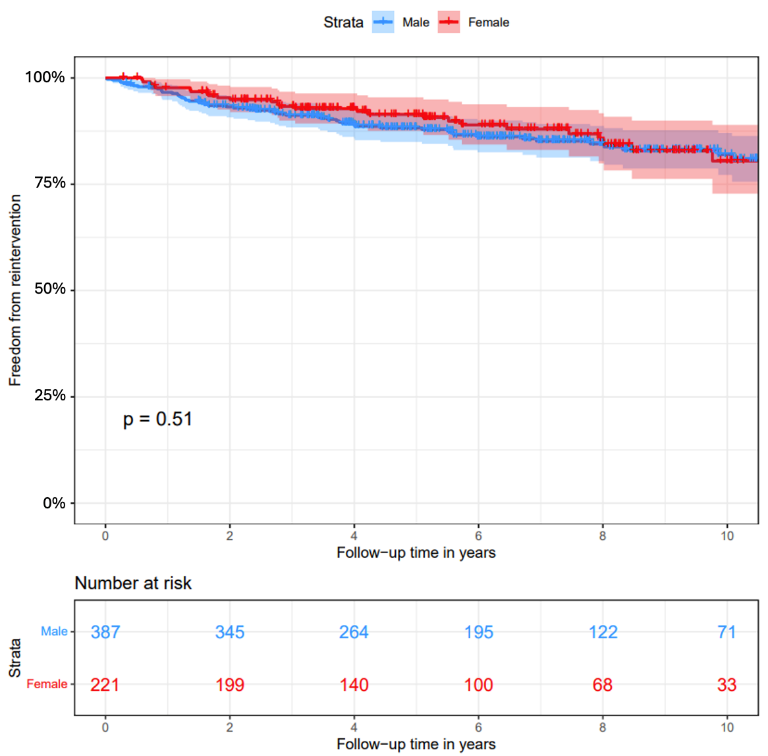

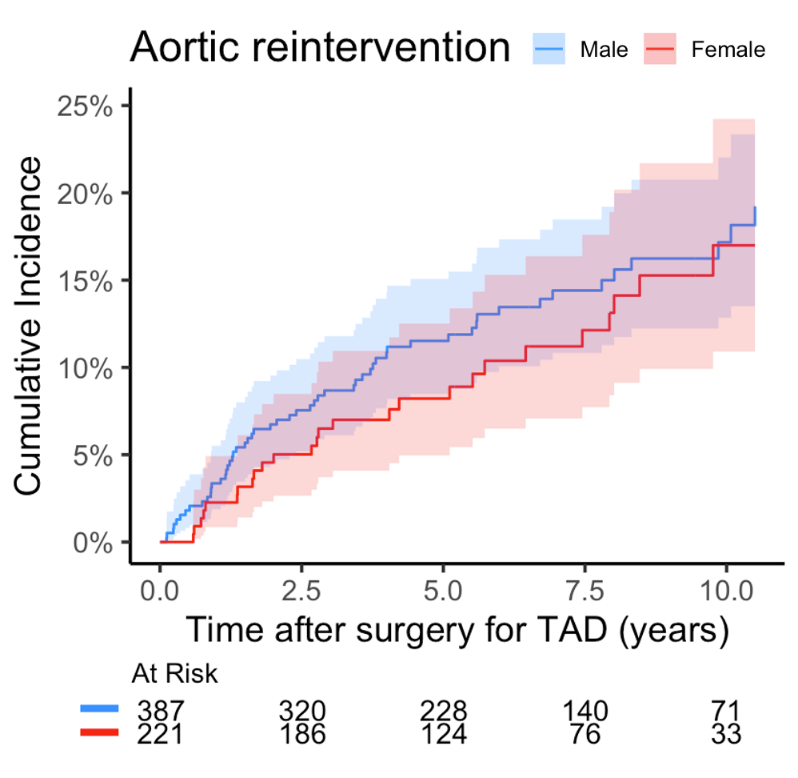


**Supplemental file 12.** All tested variables in univariable Cox proportional hazards analysis for late mortality in a complete case analysis with stratified baseline hazard.

|  | **All patients (n=711)** |  | **Females**  **(n=260)** |  | **Males**  **(n=451)** |  |
| --- | --- | --- | --- | --- | --- | --- |
| **Variable** | **HR (95%CI)** | **P value** | **HR (95%CI)** | **P value** | **HR (95%CI)** | **P value** |
| ***Patient characteristics*** |  |  |  |  |  |  |
| Female sex  *Male sex (ref)* | 1.58 (1.14-2.20) | **0.006** | - | - | - | - |
| Age (per 1 year) | 1.07 (1.06-1.09) | **<0.001** | 1.07 (1.04-1.10) | **<0.001** | 1.07 (1.04-1.09) | **<0.001** |
| History of hypertension | 1.33 (0.95-1.87) | 0.096 | 1.19 (0.70-2.02) | 0.521 | 1.34 (0.85-2.11) | 0.211 |
| History of hyperlipidaemia | 1.21 (0.72-2.04) | 0.478 | 1.26 (0.59-2.65) | 0.551 | 1.17 (0.56-2.45) | 0.670 |
| Diabetes | 1.67 (0.61-4.56) | 0.316 | 1.80 (0.55-5.85) | 0.332 | 0.92 (0.13-6.69) | 0.933 |
| COPD | 1.86 (1.05-3.30) | **0.034** | 2.61 (1.32-5.18) | **0.006** | 0.91 (0.29-2.92) | 0.879 |
| Prior CVA | 1.83 (0.96-3.51) | 0.067 | 1.44 (0.57-3.63) | 0.438 | 2.42 (0.96-6.10) | **0.060** |
| Prior MI | 2.59 (1.50-4.46) | **<0.001** | 1.85 (0.77-4.43) | 0.169 | 2.99 (1.43-6.25) | **0.004** |
| Chronic kidney disease | 3.70 (1.87-7.33) | **<0.001** | 3.35 (1.43-7.88) | **0.005** | 3.80 (1.17-12.4) | **0.027** |
| Prior TAA | 2.27 (1.34-3.83) | **0.002** | 3.30 (1.57-6.93) | **0.002** | 2.00 (0.91-4.40) | 0.083 |
| Prior aortic surgery | 3.08 (1.55-6.14) | **0.001** | 7.27 (2.52-21.0) | **<0.001** | 2.61 (1.04-6.52) | **0.041** |
| Prior cardiac surgery | 1.79 (0.96-3.33) | 0.066 | 2.39 (0.85-6.72) | 0.098 | 1.69 (0.77-3.70) | 0.192 |
| BAV | 1.30 (0.53-3.20) | 0.571 | 2.15 (0.50-9.31) | 0.307 | 1.39 (0.43-4.52) | 0.582 |
| **Surgical procedure** |  |  |  |  |  |  |
| Aortic valve surgery | 1.09 (0.75-1.59) | 0.637 | 0.75 (0.44-1.28) | 0.299 | 1.78 (1.03-3.08) | **0.038** |
| Ascending aortic surgery | 1.02 (0.25-4.16) | 0.982 | 0.82 (0.20-3.46) | 0.789 | Inf | 0.996 |
| Aortic arch surgery | 0.99 (0.67-1.47) | 0.975 | 0.85-0.50-1.46) | 0.564 | 1.20 (0.67-2.15) | 0.550 |
| Descending aortic surgery | 0.69 (0.10-5.00) | 0.717 | Inf | 0.996 | 1.24 (0.17-9.01) | 0.832 |

Univariable Cox regression analysis for all patients and females and males separately are shown. Hazard ratios and corresponding 95% confidence intervals (CI’s) are presented. P-values below 0.05 are depicted in bold. CI = confidence interval; HR = hazard ratio; Inf = infinite estimates, COPD = chronic obstructive pulmonary disease; CVA = cerebrovascular accident; MI = myocardial infarction

**Supplemental file 13.** Cox regression analysis for long term reintervention after ATAAD for all patients and stratified by sex (imputed dataset)

|  | **All patients (n=608)** |  | **Females**  **(n=221)** |  | **Males**  **(n=387)** |  |
| --- | --- | --- | --- | --- | --- | --- |
| **Variable** | **HR (95%CI)** | **P value** | **HR (95%CI)** | **P value** | **HR (95%CI)** | **P value** |
| ***Patient characteristics*** |  |  |  |  |  |  |
| Female sex  *Male sex (ref)* | 0.89 (0.55-1.44) | 0.633 | - | - | - | - |
| Age (per 1 year) | 0.98 (0.96-1.00) | 0.109 | 0.97 (0.93-1.01) | 0.098 | 0.99 (0.96-1.02) | 0.492 |
| History of hypertension | 0.69 (0.43-1.10) | 0.117 | 0.66 (0.28-1.59) | 0.341 | 0.73 (0.41-1.32) | 0.293 |
| History of hyperlipidaemia | 1.00 (0.45-2.23) | 0.991 | 0.51 (0.06-4.32) | 0.524 | 1.16 (0.48-2.79) | 0.741 |
| Diabetes | 1.01 (0.13-8.09) | 0.993 | Inf | 0.998 | 1.47 (0.19-11.5) | 0.707 |
| COPD | 0.91 (0.28-2.97) | 0.877 | 0.75 (0.09-6.27) | 0.780 | 0.98 (0.23-4.24) | 0.983 |
| Prior CVA | 0.77 (0.18-3.29) | 0.726 | 1.59 (0.34-7.52) | 0.544 | Inf | 0.996 |
| Prior MI | 0.71 (0.17-2.97) | 0.634 | Inf | 0.998 | 1.40 (0.32-6.02) | 0.650 |
| Chronic kidney disease | 0.88 (0.12-6.55) | 0.896 | 1.65 (0.19-14.2) | 0.634 | Inf | 0.996 |
| Prior TAA | 1.18 (0.36-3.90) | 0.784 | 4.74 (0.44-51.4) | 0.190 | 0.99 (0.22-4.35) | 0.985 |
| Prior aortic surgery | 2.55 (0.90-7.28) | 0.078 | 11.9 (1.15-123.5) | **0.038** | 1.99 (0.59-6.74) | 0.261 |
| Prior cardiac surgery | 0.83 (0.25-2.73) | 0.759 | 11.0 (1.26-96.3) | **0.032** | 0.51 (0.12-2.19) | 0.358 |
| BAV | 0.53 (0.07-4.00) | 0.533 | Inf | 0.998 | 0.79 (0.09-7.92) | 0.833 |
| **Surgical procedure** |  |  |  |  |  |  |
| Aortic valve surgery | 1.11 (0.66-1.88) | 0.680 | 2.08 (0.79-5.51) | 0.131 | 0.85 (0.45-1.62) | 0.612 |
| Ascending aortic surgery | 0.66 (0.09-5.04) | 0.687 | Inf | 0.997 | 0.40 (0.05-3.21) | 0.379 |
| Aortic arch surgery | 2.09 (1.13-3.86) | **0.019** | 2.46 (0.82-7.34) | 0.103 | 1.83 (0.83-4.00) | 0.129 |
| Descending aortic surgery | 2.52 (0.76-8.30) | 0.127 | 1.74 (0.20-15.2) | 0.603 | 2.84 (0.65-12.3) | 0.160 |

Univariable Cox regression analysis for all patients and females and males separately are shown. Hazard ratios and corresponding 95% confidence intervals (CI’s) are presented. P-values below 0.05 are depicted in bold. CI = confidence interval; HR = hazard ratio; Inf = infinite estimates, COPD = chronic obstructive pulmonary disease; CVA = cerebrovascular accident; MI = myocardial infarction
